# Excretable IR-820 for *in vivo* NIR-II fluorescence cerebrovascular imaging and photothermal therapy of subcutaneous tumor

**DOI:** 10.7150/thno.31332

**Published:** 2019-08-09

**Authors:** Zhe Feng, Xiaoming Yu, Minxiao Jiang, Liang Zhu, Yi Zhang, Wei Yang, Wang Xi, Gonghui Li, Jun Qian

**Affiliations:** 1 State Key Laboratory of Modern Optical Instrumentations, Centre for Optical and Electromagnetic Research; JORCEP (Sino-Swedish Joint Research Center of Photonics), Zhejiang University, Hangzhou, 310058, China; 2 Department of Urology, Sir Run-Run Shaw Hospital College of Medicine, Zhejiang University, Hangzhou 310016, China; 3 Interdisciplinary Institute of Neuroscience and Technology (ZIINT), Zhejiang University, Hangzhou, 310058, China; 4 School of Basic Medical Sciences, Zhejiang University, Hangzhou, 310058, China

**Keywords:** cyanine dye, NIR-II fluorescence, cerebrovascular imaging, photothermal therapy

## Abstract

**Rationale:** Cerebrovascular diseases, together with malignancies, still pose a huge threat to human health nowadays. With the advantages of its high spatial resolution and large penetration depth, fluorescence bioimaging in the second near-infrared spectral region (NIR-II, 900-1700 nm) and its related imaging-guided therapy based on biocompatible fluorescence dyes have become a promising theranostics method.

**Methods:** The biocompatibility of IR-820 we used in NIR-II fluorescence bioimaging was verified by long-term observation. The model of the mouse with a cranial window, the mouse model of middle cerebral artery occlusion (MCAO) and a subcutaneous xenograft mouse model of bladder tumor were established. NIR-II fluorescence cerebrovascular functional imaging was carried out by IR-820 assisted NIR-II fluorescence microscopy. Bladder tumor was treated by NIR-II fluorescence imaging-guided photothermal therapy.

**Results:** We have found that IR-820 has considerable NIR-II fluorescence intensity, and shows increased brightness in serum than in water. Herein, we achieved real time and *in vivo* cerebrovascular functional imaging of mice with high spatial resolution and large penetration depth, based on IR-820 assisted NIR-II fluorescence microscopy. In addition, IR-820 was successfully employed for NIR-II fluorescence imaging and photothermal therapy of tumor *in vivo*, and the subcutaneous tumors were inhibited obviously or eradicated completely.

**Conclusion:** Due to the considerable fluorescence intensity in NIR-II spectral region and the good photothermal effect, biocompatible and excretable IR-820 holds great potentials for functional angiography and cancer theranostics in clinical practice.

## Introduction

Second near-infrared spectral region (NIR-II) is usually defined as the wavelength range from 900 to 1700 nm 1. As an emerging and rapidly developing imaging modality, NIR-II fluorescence bioimaging has demonstrated much improvement compared to conventional NIR-I (780-900 nm) fluorescence imaging 2-11. Benefitting from low tissue scattering of emission signals, NIR-II fluorescence imaging exhibits the advantages of high spatial resolution and deep penetration 12. In addition, owing to weak tissue autofluorescence in NIR-II region, the signal-to-background ratio of NIR-II fluorescence images can be very high 13, 14. In order to obtain good imaging performance, NIR-II emissive fluorophores with certain brightness also play a vital role. So far, many kinds of probes (e.g. quantum dots (QDs) 2, 15-17, carbon nanotubes 1, 18-21 and rare-earth-doped nanocrystals 4, 22-26) have been utilized for NIR-II fluorescence whole-body and microscopic imaging. However, most of them meet with the common problem of potential biological toxicity 27-29. Organic dyes are another type of promising NIR-II contrast agents 30-38, some of which have good biocompatibility 5, 39-41. As a kind of small-molecule organic dye with good biocompatibility 42, 43, IR-820 holds potentials for clinical applications. IR-820 has been known as a commercially available NIR-I fluorescent material, since its peak emission wavelength locates in 780-900 nm. So far, there have been some reports on the surface-modification of IR-820, as well as its applications in NIR-I fluorescence bioimaging 43, 44.

Cerebrovascular disease, especially brain stroke, is one of the main leading causes of adult disability and death if not timely detected 45, 46. Currently, the main imaging modalities for the diagnosis of brain stroke in the clinical field are computerized tomography (CT) and magnetic resonance imaging (MRI), which can help identifying the location of stroke effectively 47, 48. However, both of them still have certain shortcomings, like low spatial resolution and time consuming, as well as high cost for MRI and ionizing radiation in CT 49, 50, which means they are not suitable for continuously monitoring the evolution of stroke. Thus, high-resolution and real-time optical imaging for cerebrovascular disease diagnosis has attracted tremendous scientific interests and is expected to be a low-cost and non-invasive medical modality 15, 51-57.

Malignancies are also a huge threat to human health 58-60. Traditional cancer treatment ways, including surgical removal, chemotherapy and radiotherapy, are still widely used clinically 61. However, all of them meet with several obvious side effects, such as non-specific distribution to healthy tissue, as well as drug resistance over time in chemotherapy, healthy tissue damage in radiotherapy, and the chance of relapse in surgery. Photothermal therapy (PTT), which exploits photo-induced thermal energy to realize precise, controllable and non-invasive treatment, has experienced rapid development during past few years 62-67.

In this work, we found that IR-820 showed high brightness benefitting from its large absorbance. Since IR-820 had a broad emission spectrum ranging from 700 nm to 1300 nm, it exhibited considerable NIR-II fluorescence intensity, although the peak emission wavelength fell in the NIR-I region. In addition, IR-820 showed brighter fluorescence in serum environment compared to that in water, which reveals its potential for angiography. Intravenously injected IR-820 could be excreted completely from the body of mice by their liver and kidneys after about 5 weeks, illustrating its good biocompatibility. IR-820 without any surface-modification was then utilized directly for *in vivo* NIR-II fluorescence functional cerebrovascular imaging of mice. Furthermore, with good photothermal effect under NIR-I laser excitation 42, excretable IR-820 was successfully employed for NIR-II fluorescence imaging guided photothermal therapy on subcutaneous tumor of mice. So far, few reported NIR-II photothermal probes have been proven to be excretable 68-75. So we believe that commercially available and traditional NIR-I fluorescent probe IR-820 shows great potentials to construct NIR-II fluorescence imaging based theranostic platform for clinical applications.

## Results and discussion

### Optical characterization of IR-820

**Figure 1:**
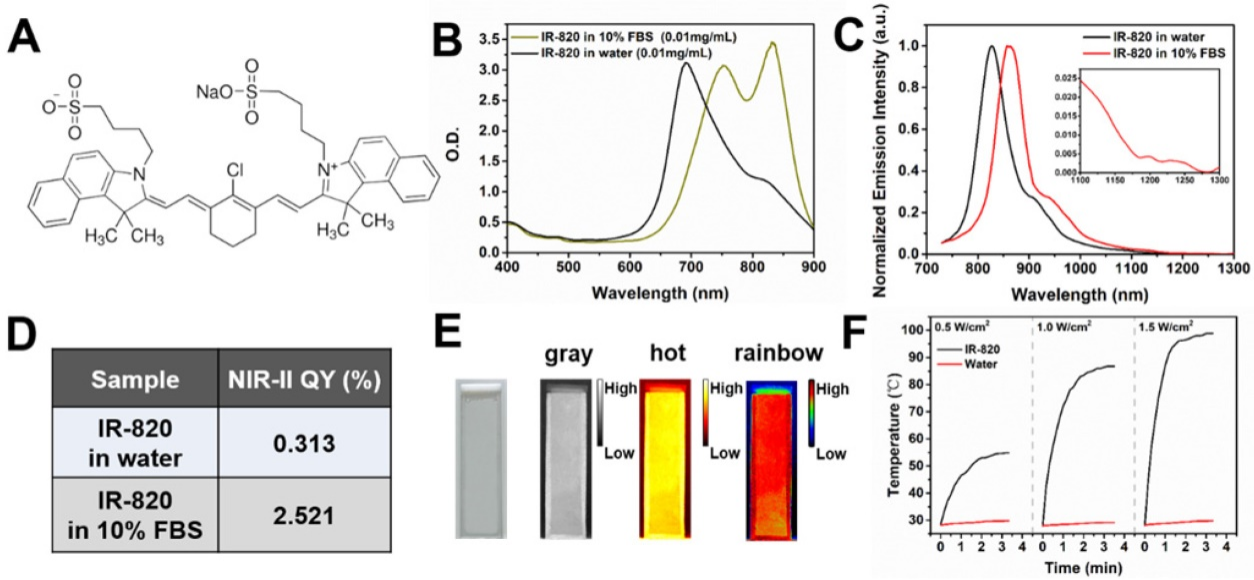
Characterization of IR-820 molecule. **(A)** Molecular structure of IR-820. **(B)** Absorption spectra of IR-820 in water and 10% FBS. The mass concentration is 0.01 mg/mL. **(C)** Normalized PL spectra of IR-820 in water and 10% FBS. The inset shows the PL spectral region beyond 1100 nm. **(D)** NIR-II quantum yields (QYs) of IR-820 in water and IR-820 in 10% FBS. **(E)** Bright-field and pseudo-color NIR-II fluorescence images of IR-820 in 10% FBS. **(F)** Heat curve of IR-820 irradiated with 793 nm laser at various power density for 10 min.

**Figure 1A** illustrates the chemical structure of IR-820, which is a kind of typical cyanine dye. As shown in **Figure 1B**, the absorption spectra of IR-820 in water and 10% fetal bovine serum (FBS) were quite different. The peak absorption wavelength of IR-820 in serum red-shifted about 143 nm, and its optical density (OD) at 793 nm was 2.505, much larger than that of IR-820 in water (1.281) when the mass concentration of IR-820 in serum and in water was the same. IR-820 in serum had the fluorescence peak at ~ 858 nm, which red-shifted about 29 nm compared to that of IR-820 in water at ~ 829 nm (**Figure 1C**). It is worth noting that the emission spectrum of IR-820 in serum could even extend to longer than 1200 nm, and its fluorescence intensity ratio in NIR-II (900-1700 nm) region was 30.17%. In addition, the quantum yield (QY) of IR-820 in serum was measured as 2.521%, about seven times higher than that of IR-820 in water (0.313%) (**Figure 1D**). It was also higher than those of many reported NIR-II fluorescent probes 76. The unique optical feature of IR-820 in serum is attributed to the fact that IR-820 molecules tend to adsorb on serum proteins and form protein-sized “nanoparticles”, which were revealed by the dynamic light scattering (DLS), Z-potential and dialysis results (**Figure S1**, **Table S1**, **Figure S2**). Binding with serum proteins could prevent IR-820 molecules form aggregates in aqueous solution, and further reduce the fluorescence self-quenching of IR-820 due to its aggregation-caused quenching (ACQ) effect. In addition, it could also promote a rigid conformation of IR-820 molecules, which minimizes the torsional rotations and result in more radiative decay (quantum yield). In addition, IR-820 exhibited good photostability when dissolved in serum (**Figure S3**), and no noticeable decrease in emission intensity was observed after continuous 793 nm laser irradiation (20 mW/cm^2^, same intensity used for the subsequent *in vivo* imaging) for 60 min (**Figure S4**). Considering IR-820 in serum had larger absorbance (at 793 nm) and fluorescence quantum yields, as well as good photostability, it is a good candidate for *in vivo* NIR-II fluorescence imaging based angiography and related applications (**Figure 1E**). IR-820 also possessed well photothermal effect. Aqueous solution of IR-820 with a fixed concentration of 500 µg/mL was irradiated with a 793 nm laser with various intensities for 10 min. At the laser intensity of 0.5 W/cm^2^, the temperature of IR-820 solution could increase to 55 ℃ within 4 min, which was already above the photothermal lethal dose (~ 49 ℃) (**Figure 1F**). Furthermore, aqueous solution of IR-820 irradiated with higher 785 nm intensity generated more prominent temperature elevation. At the laser intensity of 1.5 W/cm^2^, it even reached more than 90 ℃. In contrast, water was irradiated by the 793 nm laser with the same intensities, and the temperature elevations were negligible. The photothermal conversion efficiency of IR-820 was as high as 32.74%, which might be attributed to its large absorptivity (~ 30 L/(g×cm) ) at 793 nm, as well as the non-radiative relaxation from the excitons in the excited IR-820 molecules. The strong photothermal effect of IR-820 *in vitro* inspired us to employ them for *in vivo* photothermal therapy of tumors.

### Biodistribution and excretion of IR-820

**Figure 2:**
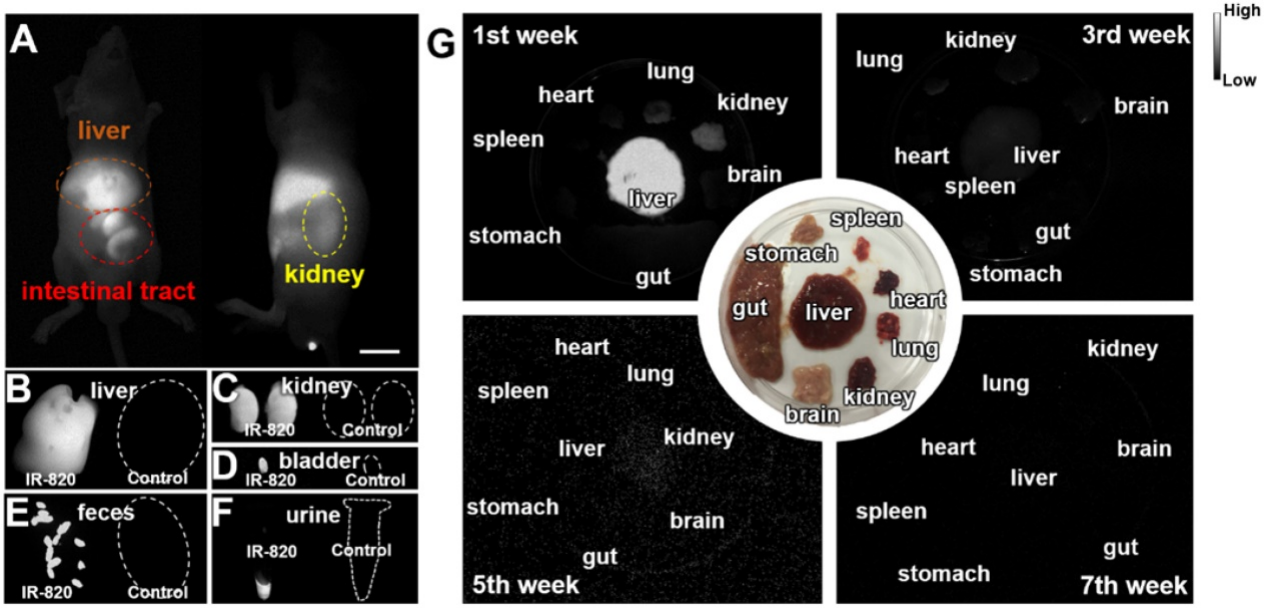
Biodistribution and excretation of IR-820 molecule. **(A)** *In vivo* NIR-II fluorescence whole-body images of mice, 48 h post the intravenous injection of IR-820 (0.5 mg/mL, 200 µL). Scale bar: 10 mm. *Ex vivo* NIR-II fluorescence images of **(B)** liver, **(C)** kidneys and **(D)** bladder harvested from the mouse 48 h post the intravenous injection of IR-820 (0.5 mg/mL, 200 µL) and PBS (1 ×, 200 µL). NIR-II fluorescence imaging of **(E)** feces and **(F)** urine collected from the IR-820 treated mouse and the PBS treated mouse before they were sacrificed. **(G)** *Ex vivo* NIR-II fluorescence images of major organs (crushed into the thin homogenate) harvested from the mice at different time points (1, 3, 5 and 7 weeks) post the intravenous injection of IR-820 (0.5 mg/mL, 200 µL). All images were taken under 793 nm laser excitation (20 mW/cm^2^). Exposure time: 50 ms.

Biocompatible and excretable fluorescent probes are significant for biological studies and clinical applications. According to the results of histology study (**Figure S5**) and blood test analysis (**Table S2-S3**), IR-820 showed negligible toxicity *in vivo*. To assess the *in vivo* kinetics of IR-820 in mice, NIR-II fluorescence imaging was conducted. Whole-body fluorescence images of mice were taken 48 h post the intravenous injection of IR-820 (0.5 mg/mL, 200 µL), and the NIR-II fluorescence intensities at the liver, kidney and gut were distinct (**Figure 2A**), indicating IR-820 localized more on these organs. The mice were then sacrificed and their organs were harvested for *ex vivo* imaging. Very strong NIR-II fluorescence signals were observed from the liver, kidney, and filled bladder (**Figure 2B**-**D**) compared to those from the control group of mice (without the treatment of IR-820). In addition, the feces and urine collected before the treated mice were sacrificed also showed brighter NIR-II fluorescence than those from the control group (**Figure 2E-F**). The *ex vivo* imaging results illustrates that IR-820 could be excreted from the body through hepatobiliary and renal system. As aforementeiond, IR-820 molecules adsorbed on serum proteins and exhibited the nanoparticle feature *in vivo*. Thus, they were excreted from body mainly through hepatobiliary system. However, the adsorption of IR-820 and serum protein should be dynamic and reversible in the mouse body. That's why small (~0.71 nm) IR-820 molecules with weak negative charge (-0.694±0.015 mV) can also be excreted via the renal system of mouse. Long-term excretion effect of IR-820 in mice body was investigated by conducting *ex vivo* NIR-II fluorescence imaging of major organs (crushed into the thin homogenate to avoid the influence caused by the thickness and optical scattering of organs) collected from the IR-820 administrated mice, at different time points (1, 3, 5 and 7 weeks) post treatment. After five weeks, almost no NIR-II fluorescence signals could be observed from all the organs (**Figure 2G**), which can help us eliminate the long-term accumulation of IR-820 in the mice body. Biocompatible and excretable IR-820 is acceptable for wide-ranging biomedical studies and applications. Especially, considering IR-820 could be excreted from mouse body through the renal system besides the hepatobiliary system, it holds the potentials for functional imaging of urinary system (e.g. urethrography and cystography).

### *In vivo* NIR-II fluorescence cerebrovascular functional imaging

**Figure 3:**
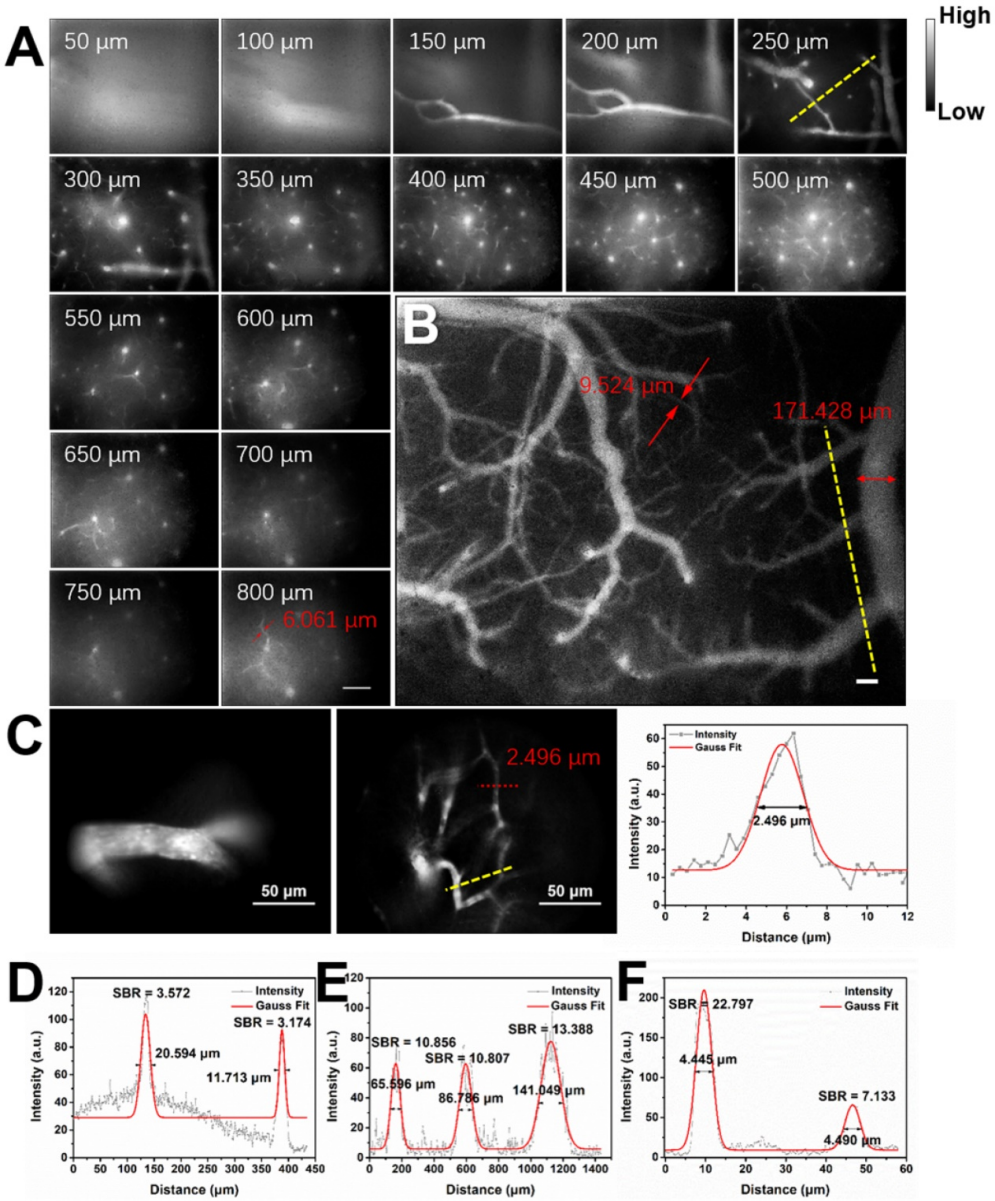
Real-time and *in vivo* NIR-II fluorescence microscopic cerebrovascular imaging of a mouse with a cranial window. **(A)** Images of blood vessels in the mouse brain at various vertical depths (0-800 μm). The mouse was intravenously injected with IR-820 (0.5 mg/mL, 200 µL). A 793 nm laser was used as the excitation and a 25× objective (XLPLN25XWMP2, 25×, NA = 1.05, Olympus) was adopted to collect the images. The red arrows show the tiny blood capillary observed at the depth of 800 μm (diameter = 6.061 μm). The frame size is 633 μm × 506 μm. Scale bar: 100 μm. **(B)** A wide-field image of brain blood vessels in the same mouse. A scan lens (LSM03, Thorlabs) was used. The frame size is 2949 μm × 2359 μm. Scale bar: 100 μm. **(C)** High-resolution images of brain blood vessels in the same mouse, at depth of 150 μm and 300 μm. A 70× endoscope objective was used. The frame size is 226 μm × 181 μm. Scale bar: 50 μm. Exposure time: 100 ms.** (D-F)** Cross-sectional fluorescence intensity profiles (gray) along the yellow lines of the cerebral blood vessels (inset of Figure 3A-C). The Gaussian fit to the profile is shown in red line in Figure 3D-F.

Considering the good NIR-II fluorescence performance of IR-820 in serum, we utilized it (0.5 mg/mL, 200 µL) for *in vivo* angiography of mice. As shown in **Figure S6**, the whole-body NIR-II fluorescence image of a nude mouse gave high spatial resolution and good clarity. The capillary with a small diameter of about 0.318 mm could be unambiguously detected and the signal to background ratio (SBR) reached 21.196. Due to low tissue scattering of NIR-II emission signals and weak tissue autofluorescence in NIR-II region, the SBR in the NIR-II fluorescence image (1200-1700 nm) was much higher than that in the NIR-I fluorescence image (800-900 nm) (**Figure S7**), though the fluorescence intensity ratio of IR-820 in 1200-1700 nm is only 0.20% while that in 800-900 nm is as high as 63.08%. However, due to the quick capture of IR-820 by the hepatobiliary and renal system, the blood vessels of mouse could hardly be seen after 10 min (**Figure S8**). To further visualize brain blood vessels, an ICR mouse with a cranial window was placed under the lab-built wide-field NIR-II fluorescence microscope for observation after intravenously injected with IR-820 (0.5 mg/mL, 200 µL). The images of the mouse brain vessels below skull were recorded (using a 25× objective, imaging speed: 9.09 frame/s) at various vertical depths, ranging from 0 μm to 800 μm. As shown in **Figure 3A**, the cerebrovascular structure was rich and could be discriminated easily. Interestingly, a capillary vessel with a small diameter of 6.061 μm was still distinguishable, even at the depth of 800 μm. Besides, an image with a large field of view was acquired by a scan lens (LSM03, Thorlabs) (**Figure 3B**), where the major blood vessels and small capillaries (diameters from 9.524 μm to 171.428 μm) could be observed clearly. It is worth mentioning that the lens is more suitable for intraoperative observation and operation when a microsurgery is performed due to its long working distance (WD = 25.1 mm). Furthermore, in order to track smaller capillaries, a 70× endoscope objective was used. As shown in **Figure 3C** and **Video S1**, a higher spatial resolution of 2.496 μm at the depth of around 300 μm was obtained, and the blood flowing in the capillary was also vividly revealed.

**Figure 4:**
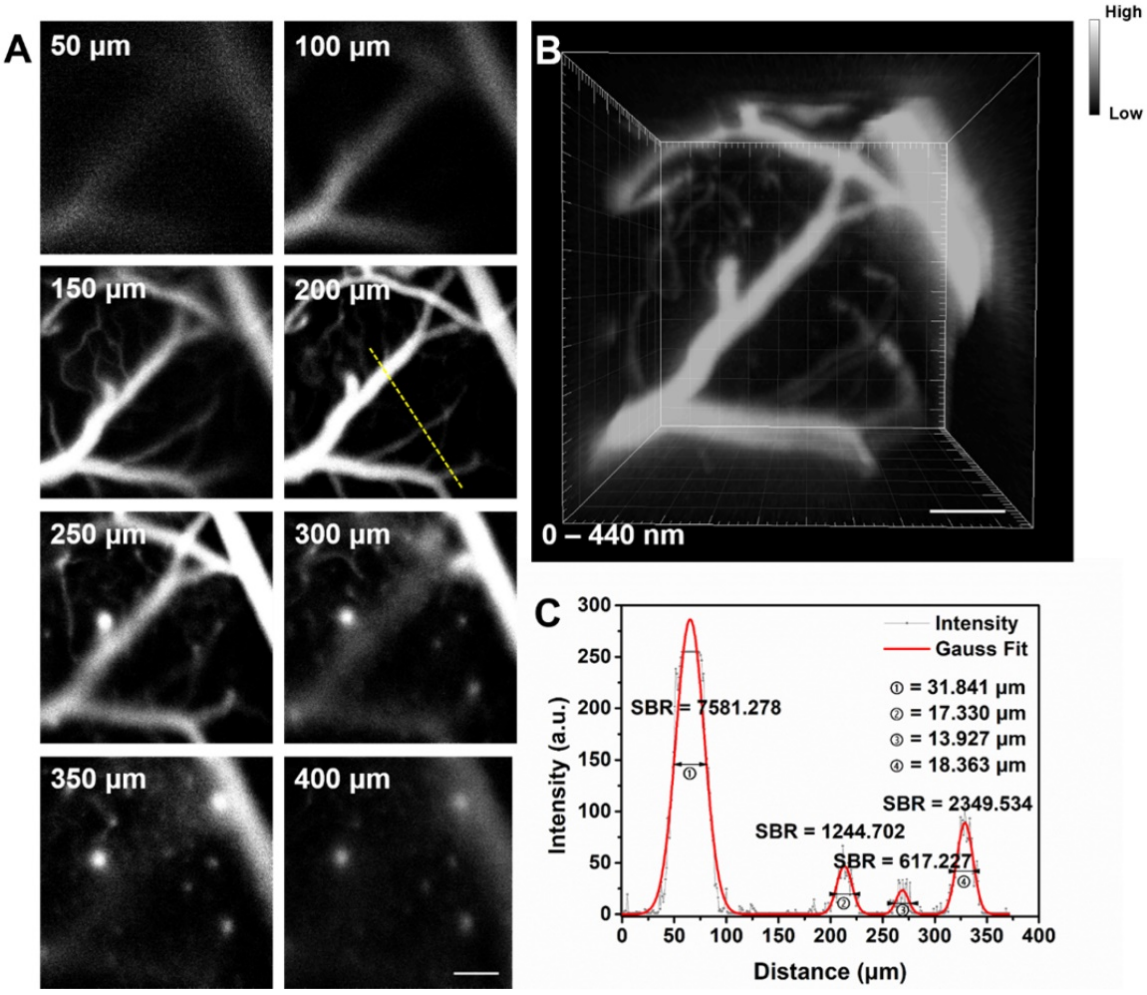
** (A)** *In vivo* NIR-II fluorescence confocal scanning microscopic images of brain vasculature of a mouse (with a cranial window) at various depths (793 nm excitation, emission >900 nm, laser power ~70 mW, PMT voltage ~600 V). **(B)** 3D reconstructed image of brain vasculatures with 400 μm depth. Scale bar: 100 μm. **(C)** A cross-sectional fluorescence intensity profile (gray) along the yellow line of the cerebral blood vessels at depth of 200 μm. The Gaussian fit to the profile is shown in red line.

Fluorescence confocal microscopy based on point-by-point excitation and single-point detection possesses the advantage of high spatial resolution, due to the existence of pinhole. Combining NIR-II fluorescence imaging with confocal microscopy is a good approach to achieve high-spatial resolution visualization of deep-tissue biosamples, as well as high-quality 3D reconstructed image. IR-820 was then utilized for NIR-II fluorescence confocal scanning microscopic cerebrovascular imaging (scanning speed: 20 μs/pixel) of mice. **Figure 4** shows the unambiguous structure of cerebral vessels at various depths below the skull. Owing to low tissue scattering of NIR-II fluorescence and weak tissue NIR-II autofluorescence, the imaging depth could reach more than 400 μm. On this basis, a 3D cerebrovascular network was acquired by reconstructing the recorded NIR-II fluorescence images at various depths.

**Figure 5:**
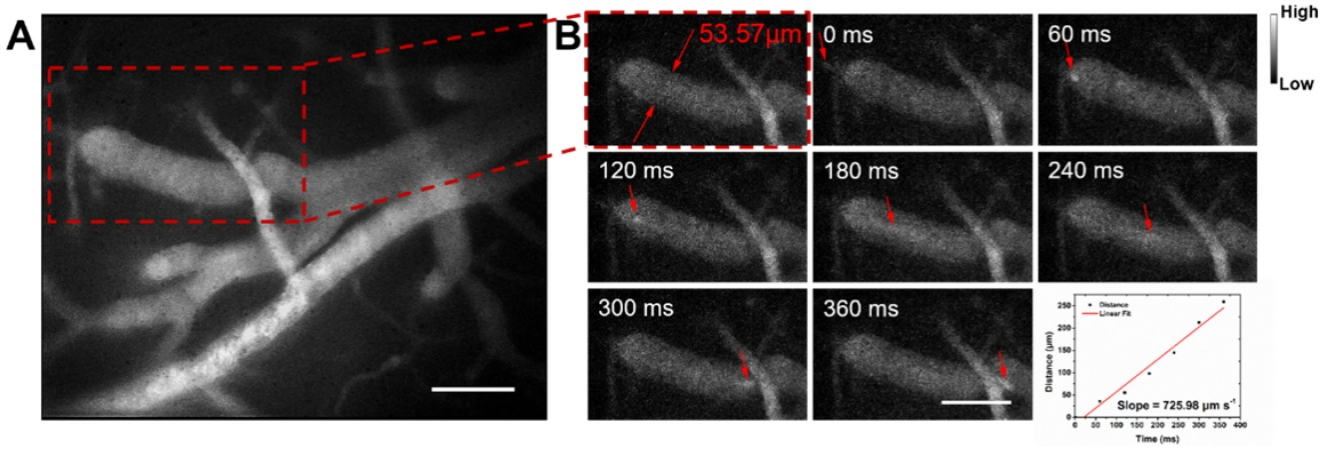
NIR-II fluorescence microscopic cerebrovascular imaging of a mouse (with a cranial window) for hemodynamics study. **(A)** A microscopic image of mouse brain vasculature at the depth of ~ 150 μm. The mouse was intravenously injected with IR-820 (0.5 mg/mL, 200 µL), and its brain was under the 793 nm laser irradiation. **(B)** The point signal at different time points was tracked in a blood capillary (diameter ≈ 53.57 μm) in the red-dashed rectangle in** (A)** and the plot of position of the point signal as a function of time. Scale bars indicate 100 µm. Objective: 25×. Exposure time: 50 ms.

Next we assessed hemodynamic characteristic in the mouse brain. The blood flowing manner in the same region (at the depth of about 150 μm below skull) was monitored at different time points with the wide-field NIR-II fluorescence microscopy. A NIR-II fluorescence video was acquired (**Video S2**), showing both the structure of the blood vessels and the real-time blood flowing (**Figure 5**). By tracking the location of a small fluorescent spot in a blood capillary (diameter ≈ 53.57 μm) in each frame (60 ms/frame) of the video, the average blood flowing velocity in the capillary was calculated about 725.98 μm/s, which met well with the normal vascular flowing speed velocity in the mouse brain (0.2-20 × 10^3^ μm/s) 1, 77, 78. It also helps us obtain the volume blood flow of 0.098 μL/min.

**Figure 6:**
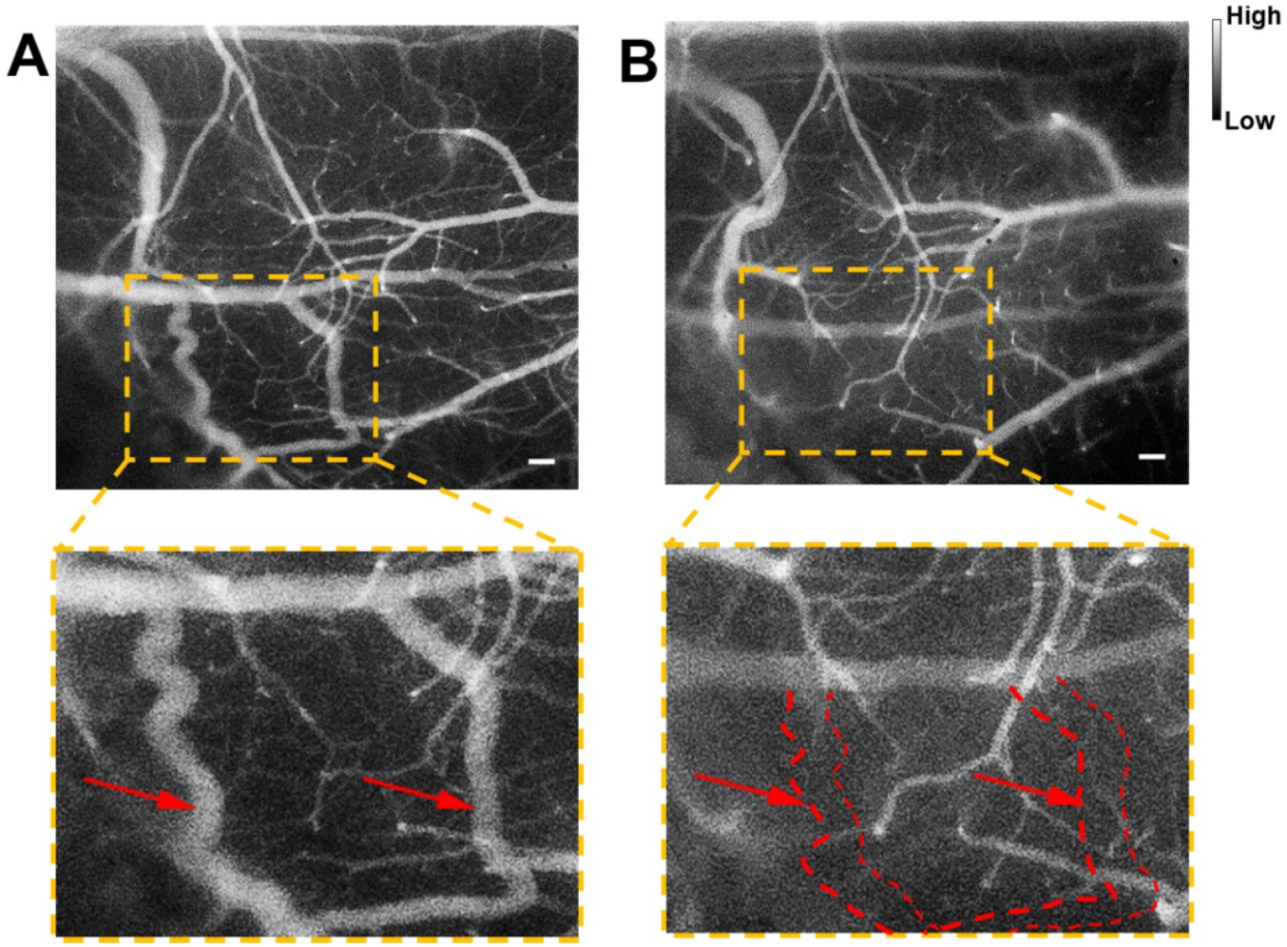
NIR-II fluorescence microscopic imaging of brain blood vessels of a mouse (with a cranial window) intravenously injected with IR-820 (0.5 mg/mL, 200 µL), before **(A)** and after **(B)** the brain thrombotic model was established. Excitation: 793 nm. Scale bar : 100 µm. Objective: scan lens (LSM03, Thorlabs). Exposure time: 100 ms.

Cerebrovascular functional imaging was further conducted with the IR-820 assisted NIR-II fluorescence microscopy. First, fluorescence imaging of an IR-820 treated ICR mouse with the cranial window was conducted, and the vascular network with a large field of view was recorded, providing rich structure information (**Figure 6A**). Several hours later, NIR-II fluorescence signals in the brain blood vessels of the mouse could not be observed, due to the hepatobiliary and renal excretion towards IR-820. Then, a microsurgery was performed on the same mouse to establish a model of middle cerebral artery occlusion (MCAO), and the mouse was imaged again with NIR-II fluorescence microscopy. According to **Figure 6B**, we found that several blood vessels in a region of right cerebral hemispherer were invisible compared with the case before the MCAO model was set up. Even in the same location, the structure and shape of blood vessels became quite different. The above results both arose from the existence of cerebral thrombosis induced by the MCAO. IR-820 with great biocompatibility can achieve diagnosis of cerebrovascular disease via high-contrast and deep-tissue NIR-II fluorescence brain angiography, holding potentials for future clinical applications.

### *In vivo* photothermal therapy of bladder tumor

**Figure 7:**
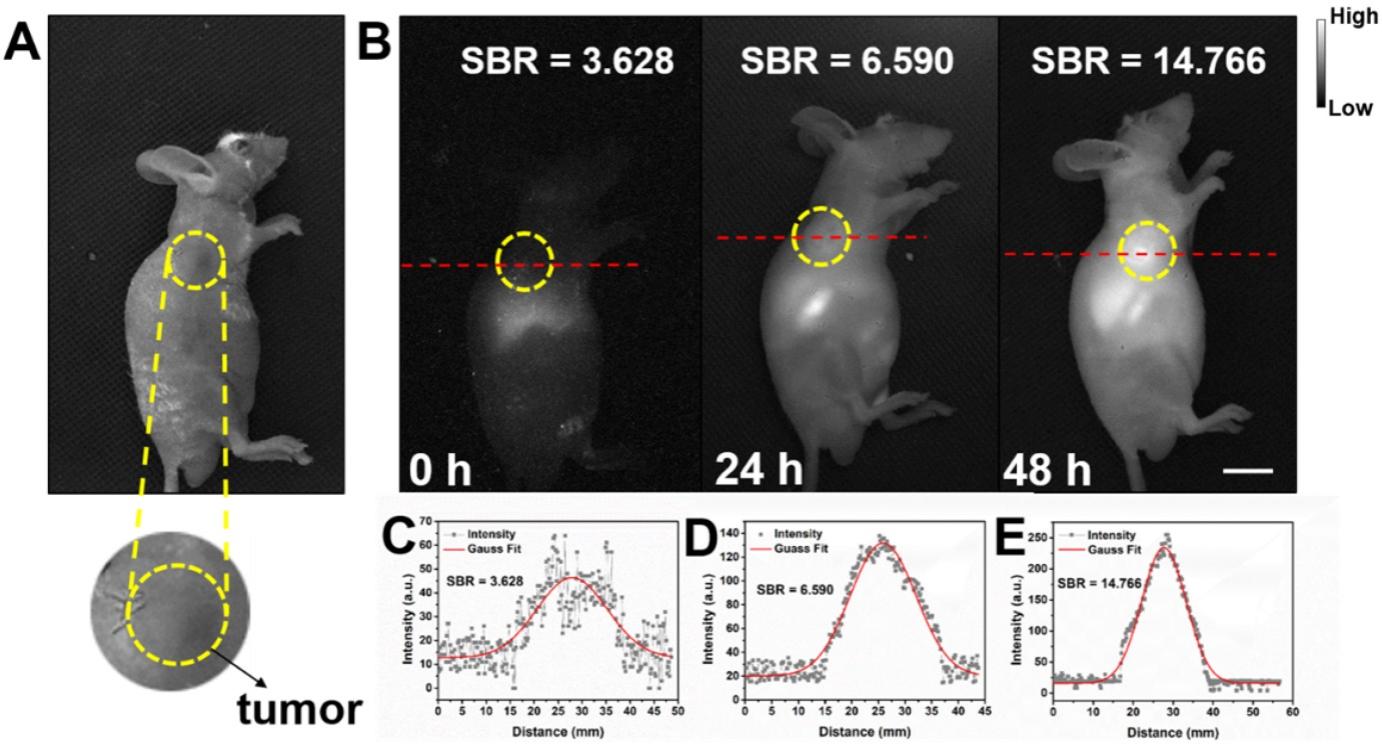
Bright field **(A)** and NIR-II fluorescence **(B)** images of a subcutaneous tumor-bearing mouse after intramuscularly injected with IR-820 (2 mg/mL, 100 µL). Intensity of 793 nm laser excitation: 20 mW/cm^2^. Exposure time: 50 ms. Scale bar: 10 mm. At **(C)** 0 h, **(D)** 24 h, and **(E)** 48 h post the injection of IR-820, a cross-sectional fluorescence intensity profiles along a red-dashed line on the mice. Gaussian fits to the profiles are shown in red lines, indicating the signal to background ratios (SBRs) defined as the ratio of NIR-II fluorescence intensity on tumor to that on the background.

Detecting tumor at an early stage is very critical for further treatment. As shown in **Figure S9**, *in vitro* NIR-II fluorescence imaging results demonstrated IR-820 could effectively stain UMUC3 cells (human bladder cancer cell line). The potential of NIR-II fluorescent IR-820 for tumor targeting *in vivo* was then studied. A subcutaneous tumor-bearing nude mouse was injected intramuscularly (from buttocks muscles) with 100 µL 1 × PBS solution of IR-820 (2 mg/mL), and imaged with the aforementioned whole-body NIR-II fluorescence imaging system. As shown in **Figure 7**, NIR fluorescence signals appeared on the site of the tumor 24 h post the treatment of IR-820. However, the SBR was not high (only 6.59). The fluorescence intensity and SBR gradually increased, and reached the maximum (14.766) at the 48th hour, indicating many IR-820 molecules have accumulated in the tumor. Thus, the time point of 48 h post treatment was selected for the follow-up *in vivo* photothermal therapy. The tumor targeting capacity of intramuscularly injected IR-820 might be attributed to its sustained release and long circulation in blood vessels, as well as related enhanced permeability and retention (EPR) effect.

**Figure 8:**
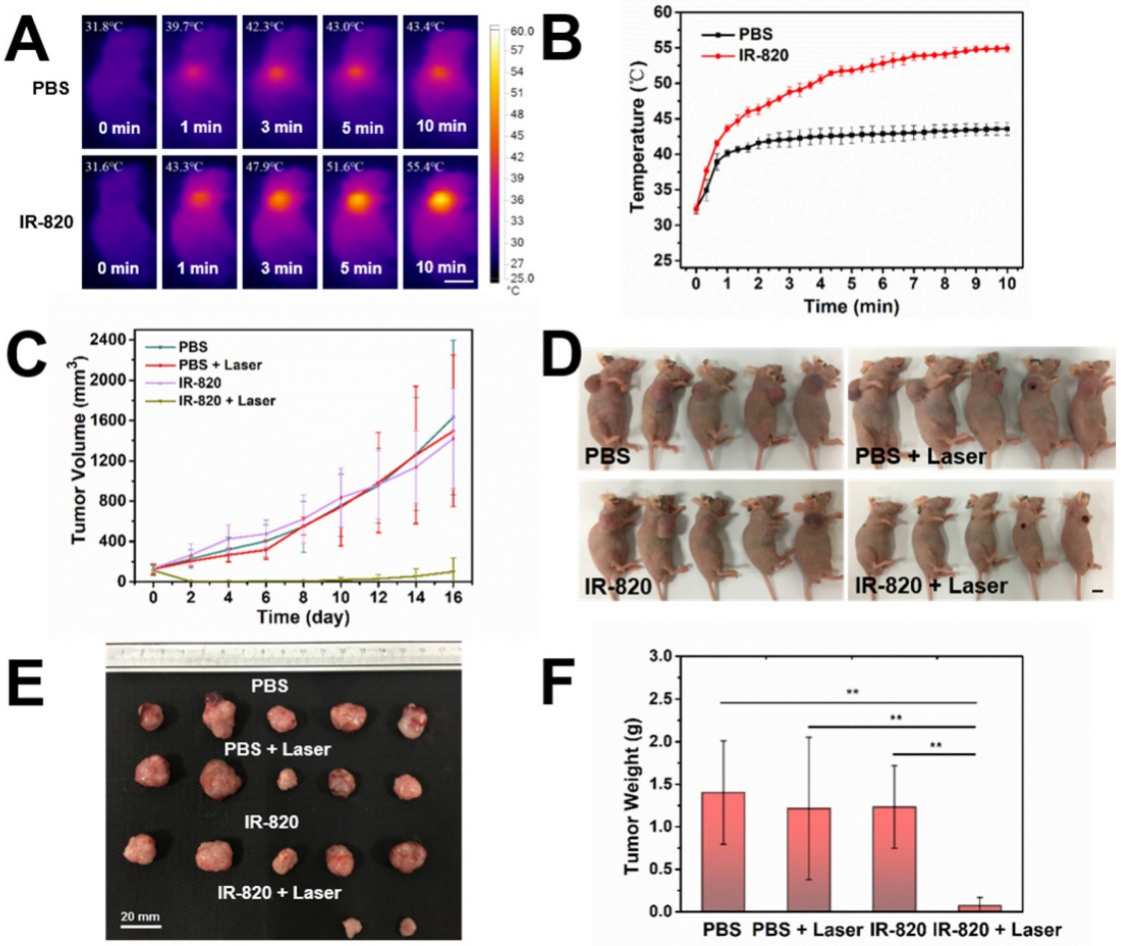
*In vivo* photothermal therapy on subcutaneous tumor-bearing mice by using IR-820. **(A)** IR thermal images of mice intramuscularly injected with PBS and PBS solution of IR-820 (2 mg/mL, 100 µL), followed by (48 h later) laser irradiation (793 nm, 2 W/cm^2^) for 1 min, 3 min, 5 min and 10 min. Scale bar: 10 mm. **(B)** Temperature changes on the subcutaneous tumor sites of the mice treated with PBS and PBS solution of IR-820, upon continuous laser irradiation. **(C)** Growth curves of the subcutaneous tumors on the mice after receiving the treatment of PBS alone, PBS & laser irradiation, IR-820 alone, and IR-820 & laser irradiation. **(D)** Images of tumor-bearing mice 16 days post various treatments. Scale bar: 10 mm. **(E)** Photographs of tumors extracted from the mice 16 days post various treatments. Scale bar: 20 mm.** (F)** Average tumor weights of mice 16 days post different treatments. n = 5, SEM, ** p ≤ 0.01.

Before using IR-820 for photothermal therapy of bladder tumor, its photothermal effect was first evaluated *in vivo*. Subcutaneous tumor-bearing mice were injected intramuscularly with PBS (100 μL) and IR-820 (2 mg/mL, 100 μL) respectively, and the tumors were then irradiated with 793 nm laser (2 W/cm^2^) 48 h post the injection. It is worth mentioning that the power density of laser irradiation would attenuate when the laser beam penetrated the skin to reach the tumor. So the actual power density for photothermal therapy of tumor should below 2 W/cm^2^. As shown in **Figure 8A-B** , after 600 s irradiation, the temperature on the tumor site of the IR-820 treated mouse rose to 55.4 ℃ (beyond the threshold cancer cells could tolerate) while that of the control mouse was only 43.4 ℃ (below the required temperature of hyperthermia). Next, twenty subcutaneous tumor-bearing mice were randomly divided into four groups, and treated with (1) PBS alone, (2) PBS and 793 nm laser irradiation, (3) IR-820 alone, and (4) IR-820 and 793 nm laser irradiation, respectively. Herein, the 793 nm laser irradiation (2 W/cm^2^, 10 min, below the maximum permissible exposure for skin in the NIR region, **Figure S10**) was performed 48 h after the treatment of PBS/IR-820 on the mice. During the next 16 days after laser irradiation, the tumor size and the body weight of the mice were monitored every other day. It could be found that only the tumors on the mice administrated with both IR-820 and 793 nm laser irradiation could be obviously inhibited (n = 2) and even cured without tumor relapse (n = 3), whereas the growth of the tumors in other three groups exhibited a similar speed instead (**Figure 8C-D** and **Figure S11A**). Besides, there were no significant differences in the average body weights of mice in all the four groups (**Figure S11B**), indicating that the IR-820 based photothermal therapy did not produced distinct side-effects on the tumor-bearing mice.

**Figure 9:**
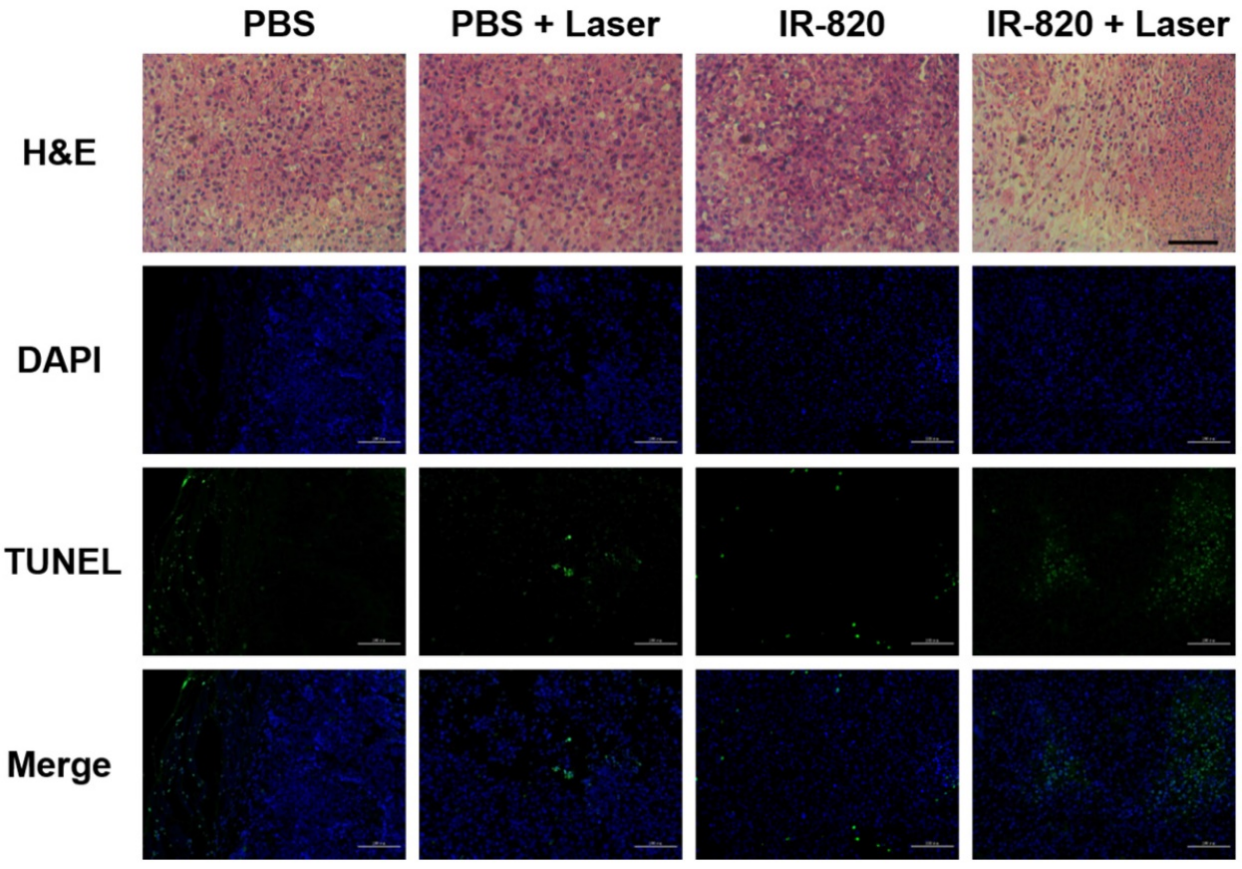
H&E and TUNEL staining analysis of tumors from mice treated with PBS, PBS + 793 nm laser irradiation, IR-820 and IR-820 + 793 nm laser irradiation. In TUNEL staining, nuclei are stained blue (DAPI staining), apoptotic cells are stained green. Images are from representative mice in each group (n = 5). Black scale bar = 50 μm, white scale bar = 100 μm.

After 16 days, all the mice were sacrificed. The tumors were separated for weighing and then sent for H&E staining and TUNEL staining. We found that IR-820 & 793 nm laser treated groups showed a significant reduction in the tumor weight compared with the mice in other three groups (P < 0.01) (**Figure 8E-F**), indicating the fact again that only IR-820 injection with laser irradiation could be beneficial to tumor inhibition and even elimination. The H&E-stained tumor section from the IR-820-treated and laser-irradiated group showed a scarlike structure containing numerous collagen fibers, compared with the three other groups. Meanwhile, the TUNEL assay result indicated the proportion of representative apoptosis-positive cells on the tumor of IR-820 & 793 nm laser treated group was obviously higher than those of other three groups (**Figure 9**).

## Conclusion

As a kind of typical NIR-I fluorescent organic small-molecule dye, IR-820 actually has broad emission ranging from 700 nm to 1300 nm, showing considerable fluorescence intensity in NIR-II spectral region. Interestingly, the fluorescence brightness of IR-820 in 10% FBS is much higher than that in water, enabling it qualified in *in vivo* NIR-II fluorescence imaging based angiography and related applications. By virtue of the bright IR-820 and NIR-II fluorescence microscopy, we conduct *in vivo* cerebrovascular imaging of mice, obtaining rich architecture of blood capillaries and achieving high spatial resolution (6.061 μm) even at the depth of ~ 800 μm. When a 70× endoscopic objective is utilized, the spatial resolution can even be improved to ~ 2.496 μm. Real-time microscopic imaging further help us get the blood flowing velocity (725.98 μm/s) in a capillary at the depth of ~ 150 μm, as well as verify the cerebral thrombosis model. IR-820 is further employed for NIR-II fluorescence imaging guided photothermal therapy. Under 793 nm laser irradiation, the subcutaneous tumors on mice can be obviously inhibited or even completely eradicated without regrowth or recurrence. Biocompatible and excretable IR-820 is promising for NIR-II fluorescence biomedical functional imaging and related clinical translation in the future.

## Methods

*Materials:* IR-820 was purchased from Shanghai Aladdin Biochemical Technology Co., Ltd.. Fetal bovine serum (FBS) was purchased from Gibco. Phosphate-buffered saline (PBS) was obtained from Sinopharm Chemical Reagent Co., Ltd., China. Deionized (DI) water prepared through an Eco-Q15 deionized water system (Shanghai Hitech Instruments Co., Ltd.) was used in all the experimental procedures unless specifically mentioned.

*General methods:* The absorption spectra were measured by a Shimadzu 2550 UV-vis scanning spectrophotometer. The photoluminescence spectra were recorded with a lab-built system based on a PG2000 spectrometer (Ideaoptics Instruments) and a 2000C spectrometer (Everuping optics Co., Ltd.) (**Figure S12**).

*Quantum yield (QY) measurements:* In order to measure the quantum yields (QYs) of IR-820 in water and IR-820 in serum, a reference of ICG with a nominal QY of 5.995% (beyond 900 nm) in DMSO was chosen 79. The absorption spectra were measured using the Shimadzu 2550 UV-vis scanning spectrophotometer and the photoluminescence (PL) measurement was carried out on a FLS980 fluorescence spectrometer (Edinburgh Instruments Ltd.). In **Figure S13**, the slopes of the straight lines describing the dependence of NIR-II fluorescence intensity upon OD (one from the reference of ICG in DMSO and the others from IR-820 in water and IR-820 in serum) were obtained. The NIR-II QY (*Q_2_*) of the sample was calculated as follows: 80


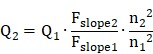


Where *Q_1_* is the QY of ICG in DMSO (5.995%, in NIR-II region), *F_slope1_* is the slope value for ICG in DMSO. *F_slope2_* is the slope value for IR-820 in water or IR-820 in serum. *n_2_* is the refractive index of water or serum, *n_1_* is the refractive index of DMSO (solvent of ICG).

*Photothermal property of IR-820:* IR-820 in aqueous solution (0.5 mg/mL, 50 μL) was irradiated by a 793 nm CW laser (Suzhou Rugkuta Optoelectronics Co., Ltd., China) with various intensities (0.5, 1.0 and 1.5 W/cm^2^) for 10 min, respectively. The temperatures were recorded every 10 s by an infrared thermal imaging camera (Ti25, Fluke IR Fusion Technology Inc., USA).

*Measurement of photothermal conversion efficiency:* To measure the photothermal conversion efficiency, IR-820 (0.1 mg/mL, 60 µL in water) in centrifuge tube was irradiated by the 793 nm laser (0.5 W/cm^2^). When the temperature of the solution raised to the maximum, the laser was switched off to cool off the solution until the room temperature. During the process, the temperature of the solution was monitored every 10 s. Finally, the photothermal conversion efficiency (η) was calculated by equation: 81


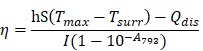


Where *h* represents the heat transfer coefficient, *S* is the container's surface area,* T_max_* is the maximum laser-triggered temperature, Tsurr is the indoor temperature, *Q_dis_* is the heat dissipation caused by the light absorbing of centrifuge tube, *I* is the power of laser (0.21 W), and *A_793_* is the absorbance of IR-820 at 793 nm. The value of *hS* can be calculated by equation:


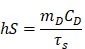


Where *m_D_* is the mass (0.06 g), *C_D_* the heat capacity of solvent (water, 4.2 J/g), and *τ_s_* is the time constant of sample system.

*Optical system for NIR-II fluorescence whole-body imaging:* As shown in **Figure S14A**, an electronic-cooling 2D (640 pixels × 512 pixels) InGaAs camera (TEKWIN SYSTEM, China) was equipped by a prime lens (focal length: 50 mm, Edmund Optics) with antireflection (AR) coating at 800-2000 nm. 793 nm laser beam was coupled to a collimator and expanded by a lens to provide uniform illumination on the irradiation area (intensity: ~ 20 mW/cm^2^), which was around 40 cm away under the camera. During the imaging, one filter (1200 nm long-pass, Edmund Optics) were utilized to extract the NIR-II fluorescence signals, as well as filter away 793 nm excitation. Owing to the good sensitivity of the camera at the spectral range of 900-1700 nm, the NIR-II fluorescence images of samples placed in the illumination area could be well recorded.

*Optical system for NIR-II fluorescence microscopic imaging:* NIR-II fluorescence microscopic imaging system was based on a commercial upright microscope (RX50, Sunny) and a 793 nm excitation laser (**Figure S14B**). After passing through a 980 nm long-pass dichroic mirror and an air objective lens (LSM03, WD = 25.1 mm, Thorlabs), an infrared transmission water-immersed object (XLPLN25XWMP2, 25×, NA = 1.05, Olympus) or a pryer endoscopic objective (70×, WD = 240 μm, NA = 0.7, Micro Control Instruments), the 793 nm laser beam illuminated onto the observed sample. NIR-II fluorescence images of the sample were collected by the same objective, and recorded with the aforementioned InGaAs camera after passing through the long-pass dichroic mirror and a 1200 nm long-pass filter (Thorlabs).

*NIR-II fluorescence confocal scanning microscope*: As shown in **Figure S15**, a 793 nm laser beam utilized as the excitation source was introduced to the confocal scanning unit and then reflected by a 900 nm long-pass dichroic mirror. Passing through a scan lens and a tube lens, the laser beam was finally focused into the sample (e.g. mouse brain) through a water-immersed object (XLPLN25XWMP2, 25×, NA = 1.05, Olympus). NIR-II fluorescence was collected with the same objective in the opposite direction, and passed through the 900 nm long-pass dichroic mirror and an optical filter (900 nm long-pass, Thorlabs). After focused into a multimode fiber, NIR-II fluorescence signals were eventually detected by an NIR (950-1700 nm) sensitive PMT (H12397-75, Hamamatsu) and amplified by an amplifier (12319, Hamamatsu). The size of the recorded image is 512 pixels × 512 pixels (scan speed is 20 µs/pixel).

*Animal preparation:* All *in vivo* experiments were performed strictly following “The National Regulation of China for Care and Use of Laboratory Animals” and approved by the Institutional Ethical Committee of Animal Experimentation of Zhejiang University. Institute of Cancer Research (ICR) mice (6-7 weeks old, female) and BALB/c nude mice (6-7 weeks old, male) were provided from the SLAC laboratory Animal Co. Ltd. (Shanghai, China) and housed in the Laboratory Animal Center of Zhejiang University (Hangzhou, China). The animal housing area was maintained at 24 ℃ with a 12 h light/dark cycle, and animals were fed with water and standard laboratory chow.

*In vitro NIR-II fluorescence cell imaging:* When UMUC3 cells were cultured in 35 mm cultivation dishes at a confluence of around 70%. 1 mL DMEM complete medium with IR-820 (10 μg/mL) was added to a UMUC3 cell plate and 1 mL DMEM complete medium without IR-820 was added to another UMUC3 cell plate as a control experiment. The cell incubation process lasted for 2 h at 37 °C and 5% CO_2_ condition. Then the cells were imaged using a wide-filed NIR-II fluorescence microscope when washed thrice with 1 × PBS.

*In vivo whole-body NIR-II fluorescence imaging:* NIR-II fluorescence images of mice were recorded, using the aforementioned NIR-II fluorescence whole-body imaging system. The anaesthetized mice were put on the platform, and intravenously injected with IR-820 (0.5 mg/mL, 200 µL). They were then immediately irradiated with the 793 nm laser (20 mW/cm^2^), and images were taken at various time points post injection.

*In vivo NIR-II fluorescence cerebrovascular imaging:* The skulls of mice were opened up through microsurgery after mice were anesthetized, and then a round thin cover glass slide was mounted onto the mouse brain and directly adhered to it through dental cement. The cover glass slide could ensure the microscopic imaging quality by flattening the mouse brain, as well as protecting it 82. The head of mouse was immobilized prior to brain angiography, and the anesthetized mouse was then intravenously injected with 200 μL 1 × PBS solution of IR-820 (0.5 mg/mL) and imaged with the aforementioned NIR-II fluorescence microscopic imaging system. Imaging was quickly conducted within a short time window (usually less than 10 min), before IR-820 was completely captured by the hepatobiliary and renal system.

*Preparation of permanent MCAO model:* Mice were anesthetized for surgery via inhalation of isoflurane. Cerebral blood flow (CBF) was determined in the area of the middle cerebral artery (MCA) by laser Doppler flowmetry. Permanent middle cerebral artery occlusion (pMCAO) model was performed in the mouse as described previously 83. Briefly, a 6-0 nylon monofilament suture, blunted at the tip and coated with 1% poly-l-lysine, was inserted 10 mm from external carotid artery into internal carotid to occlude the origin of the MCA. Animals those had less than 80% reduction in CBF in the core of the MCA area were excluded from the study. Body temperature was maintained at 37 ℃ by a heat lamp during surgery.

*Tumor models:* The UMUC3 cells (human bladder cancer cell line) were obtained from the Cell Culture Center of the Institute of Basic Medical Sciences, Chinese Academy of Medical Sciences (Shanghai, China) and cultured in the standard media recommended by American type culture collection (ATCC), and corresponding tumor models were established via the subcutaneous injection of UMUC3 cells (1×10^7^ cells in 100 μL serum-free DMEM medium) into the foreleg armpit of the male BALB/c nude mice.

*In vivo photothermal therapy:* Twenty mice were separated into 4 groups when the tumor volume reached approximately 120 mm^3^ (n = 5 per group): Mice in two groups were intramuscularly injected with PBS (100 μL), while the mice in the other two groups were intramuscularly injected with PBS dispersion of IR-820 (2 mg/mL, 100 μL). One group of PBS treated mice and one group of IR-820 treated mice were anesthetized 48 h post injection, and then irradiated by the 793 nm laser on the tumour site for 10 min, with the power density of 2 W/cm^2^. The other group of PBS treated mice and the other IR-820 treated mice were not irradiated. Infrared thermographic images and temperature changes on tumor sites were monitored by an infrared thermal imaging camera. During treatment, mice body weight and tumor sizes were measured every other day while the tumor morphologies were recorded with a cellphone every week. To measure the tumor sizes, the largest length and width of the tumors were recorded by using a vernier caliper. The tumor volume was calculated according the following formula: volume = length × width^2^ × 0.5. The tumor volume was regarded as “0” at its disappearance. In order to investigate the effect of *in vivo* photothermal therapy with IR-820, after PTT the tumors were sliced and stained with hematoxylin and eosin (H&E) and terminal deoxynucleotidyl transferase-mediated dUTP nick end labeling (TUNEL). The tissues samples were then analyzed under an inverted optical microscope and an inverted fluorescence microscope, respectively.

*Histological examinations:* Healthy female ICR mice were intravenously injected with IR-820 (0.5 mg/mL, 200 μL) and sacrificed 1 day and 28 days post the treatment. Major organs from the treated mice were collected and fixed in 10% formalin, processed routinely into paraffin sectioned, stained with hematoxylin and eosin, and imaged under an inverted optical microscope.

*Toxicological analysis:* Twelve mice were randomly divided into two groups. Each mouse in the experiment group was intravenously injected with 200 µL IR-820 solution (0.5 mg/mL in 1 × PBS) and each mouse in the control group was intravenously injected with 200 µL PBS (1 ×) solution. 1 d and 28 d post treatment, blood samples (n=3) collected from mice in the two groups were utilized for blood routine examination and hepatic & renal functions test. For each mouse, 0.3 mL blood sample was added into evacuated tubes with EDTA for blood routine examination, while another 0.6 mL blood sample was added into evacuated tubes without EDTA and then separated by centrifugation (2,000 rpm/min) for hepatic & renal functions test. Blood routine examination and hepatic & renal functions test were further conducted on a BC-2800 Vet Animal Auto Biochemistry Analyzer (MINDRAY) and an ARCHITECT C16000 (ABBOTT) Clinical Chemistry Analyzer system, respectively.

*Statistical analysis:* All results presented are mean ± standard deviation (SD). Statistical analysis was performed using Student's *t*-test. * denotes a statistical significance (* P < 0.05, ** P < 0.01, and *** P < 0.001) between the experience data of two groups.

## Abbreviations

AR: antireflection
CBF: cerebral blood flow
CT: computerized tomography
CW: continuous wave
DI: deionized
DMEM: dulbecco's modified eagle mediumE
EPR: enhanced permeability and retention
FBS: fetal bovine serum
H&E: hematoxylin-eosin
ICR: institute of cancer research
MCAO: middle cerebral artery occlusion
MRI: magnetic resonance imaging
NA: numerical aperture
NIR: near infrared region
OD: optical density
PBS: phosphate-buffered saline
PTT: photothermal therapy
QDs: quantum dots
QY: quantum yields
SBR: signal to background ratio
SD: standard deviation
TUNEL: terminal deoxynucleotidyl transferase-mediated dUTP nick end labeling
WD: working distance

## References

[1] Hong G, Lee JC, Robinson JT, Raaz U, Xie L, Huang NF (2012). Multifunctional *in vivo* vascular imaging using near-infrared II fluorescence. Nat Med.

[2] Hong GS, Robinson JT, Zhang YJ, Diao S, Antaris AL, Wang QB (2012). *In vivo* fluorescence imaging with Ag2S quantum dots in the second near-infrared region. Angew Chem Int Ed Engl.

[3] Hong G, Zou Y, Antaris AL, Diao S, Wu D, Cheng K (2014). Ultrafast fluorescence imaging *in vivo* with conjugated polymer fluorophores in the second near-infrared window. Nat Commun.

[4] Naczynski DJ, Tan MC, Zevon M, Wall B, Kohl J, Kulesa A (2013). Rare-earth-doped biological composites as *in vivo* shortwave infrared reporters. Nat Commun.

[5] Carr JA, Franke D, Caram JR, Perkinson CF, Saif M, Askoxylakis V (2018). Shortwave infrared fluorescence imaging with the clinically approved near-infrared dye indocyanine green. Proc Natl Acad Sci U S A.

[6] Tang Y, Li Y, Wang Z, Pei F, Hu X, Ji Y (2018). Organic semiconducting nanoprobe with redox-activatable NIR-II fluorescence for *in vivo* real-time monitoring of drug toxicity. Chem Commun (Camb).

[7] Sun Y, Zeng X, Xiao Y, Liu C, Zhu H, Zhou H (2018). Novel dual-function near-infrared II fluorescence and PET probe for tumor delineation and image-guided surgery. Chem Sci.

[8] Ding F, Zhan YB, Lu XJ, Sun Y (2018). Recent advances in near-infrared II fluorophores for multifunctional biomedical imaging. Chem Sci.

[9] He SQ, Song J, Qu JL, Cheng Z (2018). Crucial breakthrough of second near-infrared biological window fluorophores: design and synthesis toward multimodal imaging and theranostics. Chem Soc Rev.

[10] Xu YL, Tian M, Zhang H, Xiao YL, Hong XC, Sun Y (2018). Recent development on peptide-based probes for multifunctional biomedical imaging. Chin Chem Lett.

[11] Yang J, Xie Q, Zhou H, Chang L, Wei W, Wang Y (2018). Proteomic analysis and NIR-II imaging of MCM2 protein in hepatocellular carcinoma. J Proteome Res.

[12] Bashkatov AN, Genina EA, Kochubey VI, Tuchin VV (2005). Optical properties of human skin, subcutaneous and mucous tissues in the wavelength range from 400 to 2000 nm. J Phys D Appl Phys.

[13] Del Rosal B, Villa I, Jaque D, Sanz-Rodriguez F (2016). *In vivo* autofluorescence in the biological windows: the role of pigmentation. J Biophotonics.

[14] Diao S, Hong G, Antaris AL, Blackburn JL, Cheng K, Cheng Z (2015). Biological imaging without autofluorescence in the second near-infrared region. Nano Res.

[15] Zebibula A, Alifu N, Xia L, Sun C, Yu X, Xue D (2018). Ultrastable and biocompatible NIR-II quantum dots for functional bioimaging. Adv Funct Mater.

[16] Dong B, Li C, Chen G, Zhang Y, Zhang Y, Deng M (2013). Facile synthesis of highly photoluminescent Ag2Se quantum dots as a new fluorescent probe in the second near-infrared window for *in vivo* imaging. Chem Mater.

[17] Sasaki A, Tsukasaki Y, Komatsuzaki A, Sakata T, Yasuda H, Jin T (2015). Recombinant protein (EGFP-Protein G)-coated PbS quantum dots for *in vitro* and *in vivo* dual fluorescence (visible and second-NIR) imaging of breast tumors. Nanoscale.

[18] Diao S, Hong G, Robinson JT, Jiao L, Antaris AL, Wu JZ (2012). Chirality enriched (12,1) and (11,3) single-walled carbon nanotubes for biological imaging. J Am Chem Soc.

[19] Hong G, Diao S, Chang J, Antaris AL, Chen C, Zhang B (2014). Through-skull fluorescence imaging of the brain in a new near-infrared window. Nat Photonics.

[20] Robinson JT, Hong GS, Liang YY, Zhang B, Yaghi OK, Dai HJ (2012). *In vivo* fluorescence imaging in the second near-infrared window with long circulating carbon nanotubes capable of ultrahigh tumor uptake. J Am Chem Soc.

[21] Welsher K, Sherlock SP, Dai H (2011). Deep-tissue anatomical imaging of mice using carbon nanotube fluorophores in the second near-infrared window. Proc Natl Acad Sci U S A.

[22] Yang Y, Wang P, Lu L, Fan Y, Sun C, Fan L (2018). Small-molecule lanthanide complexes probe for second near-infrared window bioimaging. Anal Chem.

[23] Wang R, Li X, Zhou L, Zhang F (2014). Epitaxial seeded growth of rare-earth nanocrystals with efficient 800 nm near-infrared to 1525 nm short-wavelength infrared downconversion photoluminescence for *in vivo* bioimaging. Angew Chem Int Ed Engl.

[24] Dang X, Gu L, Qi J, Correa S, Zhang G, Belcher AM (2016). Layer-by-layer assembled fluorescent probes in the second near-infrared window for systemic delivery and detection of ovarian cancer. Proc Natl Acad Sci U S A.

[25] Liu L, Wang SF, Zhao BZ, Pei P, Fan Y, Li XM (2018). Er3+ sensitized 1530nm to 1180 nm second near-infrared window upconversion nanocrystals for invivo biosensing. Angew Chem Int Ed Engl.

[26] Wang PY, Fan Y, Lu LF, Liu L, Fan LL, Zhao MY (2018). NIR-II nanoprobes in-vivo assembly to improve image-guided surgery for metastatic ovarian cancer. Nat Commun.

[27] Wang Y, Hu R, Lin G, Roy I, Yong KT (2013). Functionalized quantum dots for biosensing and bioimaging and concerns on toxicity. ACS Appl Mater Interfaces.

[28] Alshehri R, Ilyas AM, Hasan A, Arnaout A, Ahmed F, Memic A (2016). Carbon nanotubes in biomedical applications: factors, mechanisms, and remedies of toxicity. J Med Chem.

[29] Tian J, Zeng X, Xie X, Han S, Liew O-W, Chen Y-T (2015). Intracellular adenosine triphosphate deprivation through lanthanide-doped nanoparticles. J Am Chem Soc.

[30] Tang YF, Li YY, Hu XM, Zhao H, Ji Y, Chen L (2018). "Dual Lock-and-Key"-controlled nanoprobes for ultrahigh specific fluorescence imaging in the second near-infrared window. Adv Mater.

[31] Zhu SJ, Hu ZB, Tian R, Yung BC, Yang QL, Zhao S (2018). Repurposing cyanine NIR-I dyes accelerates clinical translation of near-infrared-II (NIR-II) bioimaging. Adv Mater.

[32] Sun Y, Ding M, Zeng X, Xiao Y, Wu H, Zhou H (2017). Novel bright-emission small-molecule NIR-II fluorophores for *in vivo* tumor imaging and image-guided surgery. Chem Sci.

[33] Sun Y, Qu CR, Chen H, He MM, Tang C, Shou KQ (2016). Novel benzo-bis(1,2,5-thiadiazole) fluorophores for *in vivo* NIR-II imaging of cancer. Chem Sci.

[34] Zhou H, Xiao YL, Hong XC (2018). New NIR-II dyes without a benzobisthiadiazole core. Chin Chem Lett.

[35] Antaris AL, Chen H, Cheng K, Sun Y, Hong G, Qu C (2016). A small-molecule dye for NIR-II imaging. Nat Mater.

[36] Antaris AL, Chen H, Diao S, Ma ZR, Zhang Z, Zhu SJ (2017). A high quantum yield molecule-protein complex fluorophore for near-infrared II imaging. Nat Commun.

[37] Zhu SJ, Yung BC, Chandra S, Niu G, Antaris AL, Chen XY (2018). Near-infrared-II (NIR-II) bioimaging via off-peak NIR-I fluorescence emission. Theranostics.

[38] Lin JC, Zeng XD, Xiao YL, Tang L, Nong JX, Liu YF (2019). Novel near-infrared II aggregation-induced emission dots for *in vivo* bioimaging. Chem Sci.

[39] Alford R, Simpson HM, Duberman J, Hill GC, Ogawa M, Regino C (2009). Toxicity of organic fluorophores used in molecular imaging: literature review. Mol Imaging.

[40] Starosolski Z, Bhavane R, Ghaghada KB, Vasudevan SA, Kaay A, Annapragada A (2017). Indocyanine green fluorescence in second near-infrared (NIR-II) window. PLoS One.

[41] Bhavane R, Starosolski Z, Stupin I, Ghaghada KB, Annapragada A (2018). NIR-II fluorescence imaging using indocyanine green nanoparticles. Sci Rep.

[42] Fernandez-Fernandez A, Manchanda R, Lei T, Carvajal DA, Tang Y, Kazmi SZR (2012). Comparative study of the optical and heat generation properties of IR820 and indocyanine green. Mol Imaging.

[43] Prajapati SI, Martinez CO, Bahadur AN, Wu IQ, Zheng W, Lechleiter JD (2009). Near-infrared imaging of injured tissue in living subjects using IR-820. Mol Imaging.

[44] Qian J, Wang D, Cai F, Zhan Q, Wang Y, He S (2012). Photosensitizer encapsulated organically modified silica nanoparticles for direct two-photon photodynamic therapy and *in vivo* functional imaging. Biomaterials.

[45] Pagan E, Chatenoud L, Rodriguez T, Bosetti C, Levi F, Malvezzi M (2017). Comparison of trends in mortality from coronary heart and cerebrovascular diseases in North and South America: 1980 to 2013. Am J Cardiol.

[46] Feigin VL, Forouzanfar MH, Krishnamurthi R, Mensah GA, Connor M, Bennett DA (2014). Journal of photochemistry & photobiology c photochemistry reviews. Lancet.

[47] Ogawa S, Lee TM, Kay AR, Tank DW (1990). Brain magnetic-resonance-imaging with contrast dependent on blood oxygenation. Proc Natl Acad Sci U S A.

[48] Alberts MJ, Latchaw RE, Jagoda A, Wechsler LR, Crocco T, George MG (2011). Revised and updated recommendations for the establishment of primary stroke centers: a summary statement from the brain attack coalition. Stroke.

[49] Myles P, Evans S, Lophatananon A, Dimitropoulou P, Easton D, Key T (2008). Diagnostic radiation procedures and risk of prostate cancer. Br J Cancer.

[50] Brenner DJ, Hall EJ (2007). Current concepts - computed tomography - an increasing source of radiation exposure. N Engl J Med.

[51] Qi J, Sun C, Zebibula A, Zhang H, Kwok RTK, Zhao X (2018). Real-time and high-resolution bioimaging with bright aggregation-induced emission dots in short-wave infrared region. Adv Mater.

[52] Zhang XD, Wang HS, Antaris AL, Li LL, Diao S, Ma R (2016). Traumatic brain injury imaging in the second near-infrared window with a molecular fluorophore. Adv Mater.

[53] Wang YL, Hu RR, Xi W, Cai FH, Wang SW, Zhu ZF (2015). Red emissive AIE nanodots with high two-photon absorption efficiency at 1040 nm for deep-tissue *in vivo* imaging. Biomed Opt Express.

[54] Ding F, Li C, Xu Y, Li J, Li H, Yang G (2018). PEGylation regulates self-assembled small-molecule dye-based probes from single molecule to nanoparticle size for multifunctional NIR-II bioimaging.

[55] Zhen SJ, Wang SW, Li SW, Luo WW, Gao M, Ng LG (2018). Efficient red/near-infrared fluorophores based on benzo[1,2-b:4,5-b ']dithiophene 1,1,5,5-tetraoxide for targeted photodynamic therapy and *in vivo* two-photon fluorescence bioimaging. Adv Funct Mater.

[56] Sheng ZH, Guo B, Hu DH, Xu SD, Wu WB, Liew WH (2018). Bright aggregation-induced-emission dots for targeted synergetic NIR-II fluorescence and NIR-I photoacoustic imaging of orthotopic brain tumors. Adv Mater.

[57] Li BH, Lu LF, Zhao MY, Lei ZH, Zhang F (2018). An Efficient 1064 nm NIR-II excitation fluorescent molecular dye for feep-tissue high-resolution dynamic bioimaging. Angew Chem Int Ed Engl.

[58] Torre LA, Bray F, Siegel RL, Ferlay J, Lortet-Tieulent J, Jemal A (2015). Global cancer statistics, 2012. CA Cancer J Clin.

[59] Lopez-Gomez M, Malmierca E, de Gorgolas M, Casado E (2013). Cancer in developing countries: The next most preventable pandemic. The global problem of cancer. Crit Rev Oncol Hematol.

[60] Vineis P, Wild CP (2014). Global cancer patterns: causes and prevention. Lancet.

[61] Miller KD, Siegel RL, Lin CC, Mariotto AB, Kramer JL, Rowland JH (2016). Cancer treatment and survivorship statistics, 2016. CA Cancer J Clin.

[62] Wang S, Zhao X, Wang S, Qian J, He S (2016). Biologically inspired polydopamine capped gold nanorods for drug delivery and light-mediated cancer therapy. ACS Appl Mater Interfaces.

[63] Yang T, Tang Y, Liu L, Lv X, Wang Q, Ke H (2017). Size-dependent Ag2S nanodots for second near-Infrared fluorescence/photoacoustics imaging and simultaneous photothermal therapy. ACS Nano.

[64] Alves CG, Lima-Sousa R, de Melo-Diogo D, Louro RO, Correia IJ (2018). IR780 based nanomaterials for cancer imaging and photothermal, photodynamic and combinatorial therapies. Int J Pharm.

[65] Shibu ES, Hamada M, Murase N, Biju V (2013). Nanomaterials formulations for photothermal and photodynamic therapy of cancer. J Photochem Photobiol C Photochem Rev.

[66] Zeng XD, Xiao YL, Lin JC, Li SS, Zhou H, Nong JX (2018). Near-infrared II dye-protein complex for biomedical imaging and imaging-guided photothermal therapy. Adv Healthc Mater.

[67] Alifu N, Zebibula A, Qi J, Zhang HQ, Sun CW, Yu XM (2018). Single-molecular near-infrared-II theranostic systems: ultrastable aggregation-induced emission nanoparticles for long-term tracing and efficient photothermal therapy. ACS Nano.

[68] Li XL, Jiang MY, Zeng SJ, Liu HR (2019). Polydopamine coated multifunctional lanthanide theranostic agent for vascular malformation and tumor vessel imaging beyond 1500 nm and imaging-guided photothermal therapy. Theranostics.

[69] Zhao DH, Yang J, Xia RX, Yao MH, Jin RM, Zhao YD (2018). High quantum yield Ag_2_S quantum dot@polypeptide-engineered hybrid nanogels for targeted second near-infrared fluorescence/photoacoustic imaging and photothermal therapy. Chem Commun (Camb).

[70] Hu XM, Tang YF, Hu YX, Lu F, Lu XM, Wang YQ (2019). Gadolinium-chelated conjugated polymer-based nanotheranostics for photoacoustic/magnetic resonance/NIR-II fluorescence imaging-guided cancer photothermal therapy. Theranostics.

[71] Wang H, Mu QX, Wang K, Revia RA, Yen C, Gu XY (2019). Nitrogen and boron dual-doped graphene quantum dots for near-infrared second window imaging and photothermal therapy. Appl Mater Today.

[72] Zeng XD, Xiao YL, Lin JC, Li SS, Zhou H, Nong JX (2018). Near-infrared II dye-protein complex for biomedical imaging and imaging-guided photothermal therapy. Adv Healthc Mater.

[73] Li TW, Li CY, Ruan Z, Xu PP, Yang XH, Yuan P (2019). Polypeptide-conjugated second near-infrared organic fluorophore for image-guided photothermal therapy. ACS Nano.

[74] Miao YW, Gu CT, Yu B, Zhu YW, Zou WT, Shen YQ (2019). A conjugated polymer-based nanoparticles with efficient NIR-II fluorescent, photoacoustic and photothermal performance. Chembiochem.

[75] Shi B, Yan QL, Tang J, Xin K, Zhang JC, Zhu Y (2018). Hydrogen Sulfide-Activatable Second Near-Infrared Fluorescent Nanoassemblies for Targeted Photothermal Cancer Therapy. Nano Lett.

[76] Hong GS, Antaris AL, Dai HJ (2017). Near-infrared fluorophores for biomedical imaging. Nat biomed Eng.

[77] Barretto RP, Ko TH, Jung JC, Wang TJ, Capps G, Waters AC (2011). Time-lapse imaging of disease progression in deep brain areas using fluorescence microendoscopy. Nat Med.

[78] Guo B, Feng Z, Hu DH, Xu SD, Middha E, Pan YT (1902). Precise deciphering of brain vasculatures and microscopic tumors with dual NIR-II fluorescence and photoacoustic imaging.

[79] Benson RC, Kues HA (1977). Absorption and fluorescence properties of cyanine dyes. J Chem Eng Data.

[80] Williams ATR, Winfield SA, Miller JN (1983). Relative fluorescence quantum yields using a computer-controlled luminescence spectrometer. Analyst.

[81] Roper DK, Ahn W, Hoepfner M (2007). Microscale heat transfer transduced by surface plasmon resonant gold nanoparticles. J Phys Chem C Nanomater Interfaces.

[82] Qian J, Wang D, Cai FH, Xi W, Peng L, Zhu ZF (2012). Observation of multiphoton-induced fluorescence from graphene oxide nanoparticles and applications in *in vivo* functional bioimaging. Angew Chem Int Ed Engl.

[83] Ansari S, Azari H, McConnell DJ, Afzal A, Mocco J (2011). Intraluminal middle cerebral artery occlusion (MCAO) model for ischemic stroke with laser doppler flowmetry guidance in mice. J Vis Exp.

