# Excretable IR-820 for *in vivo* NIR-II fluorescence cerebrovascular imaging and photothermal therapy of subcutaneous tumor: Erratum

**DOI:** 10.7150/thno.78444

**Published:** 2022-11-02

**Authors:** Zhe Feng, Xiaoming Yu, Minxiao Jiang, Liang Zhu, Yi Zhang, Wei Yang, Wang Xi, Gonghui Li, Jun Qian

**Affiliations:** 1 State Key Laboratory of Modern Optical Instrumentations, Centre for Optical and Electromagnetic Research; JORCEP (Sino-Swedish Joint Research Center of Photonics), Zhejiang University, Hangzhou, 310058, China; 2 Department of Urology, Sir Run-Run Shaw Hospital College of Medicine, Zhejiang University, Hangzhou 310016, China; 3 Interdisciplinary Institute of Neuroscience and Technology (ZIINT), Zhejiang University, Hangzhou, 310058, China; 4 School of Basic Medical Sciences, Zhejiang University, Hangzhou, 310058, China

One microscopic image of tissue sections of the mouse brain in Fig. S5 (Supplementary Files) was chosen incorrectly due to wrong data naming. We have performed the histological examination again to ensure scientific rigor in this work. The newly arranged Fig. S5 is as follows. Notably, the correction does not affect the main conclusion of our paper. We genuinely apologize to the Editor and the readership of the journal for any inconvenience it caused.

## Figures and Tables

**Figure S5:**
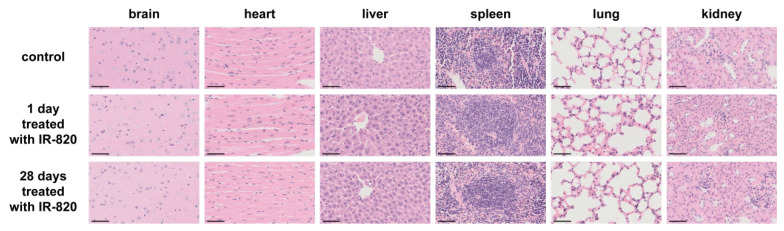
Microscopic images of tissue sections from mice injected with 1 × PBS solution (200 μL) as control, PBS solution of IR-820 (0.5 mg/mL, 200 µL) for 1 day and 28 days respectively. Scale bar: 50 μm.

