# Supplementary material for: Excretable IR-820 for in vivo NIR-II fluorescence cerebrovascular imaging and photothermal therapy of subcutaneous tumor
Source: Theranostics. 2019 Aug 9;9(19):5706–19. doi: 10.7150/thno.31332 (PMC6735390; doi:10.7150/thno.31332)
Supplement: Supplementary file 1 — Supplementary figures and tables. [file thnov09p5706s1.pdf]

Supplementary Files

Excretable IR-820 for *in vivo* NIR-II fluorescence cerebrovascular imaging and photothermal therapy of subcutaneous tumor

*Zhe Feng#, Xiaoming Yu#, Minxiao Jiang, Liang Zhu, Yi Zhang, Wei Yang, Wang Xi, Gonghui Li, Jun Qian**

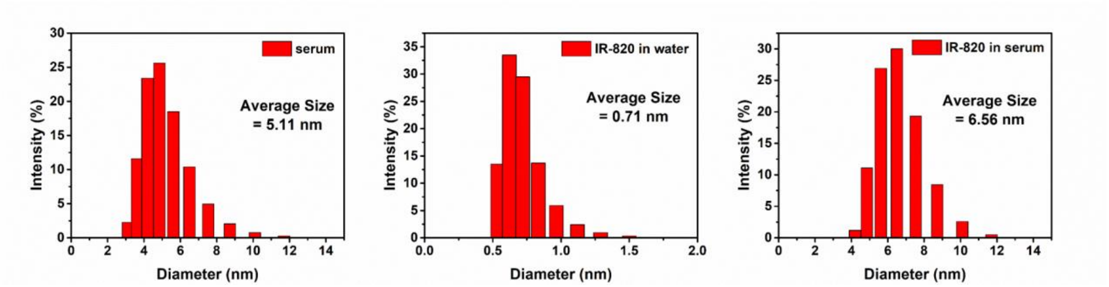

Figure S1. Representative DLS results of serum solution, IR-820 in water, and IR-820 in serum.

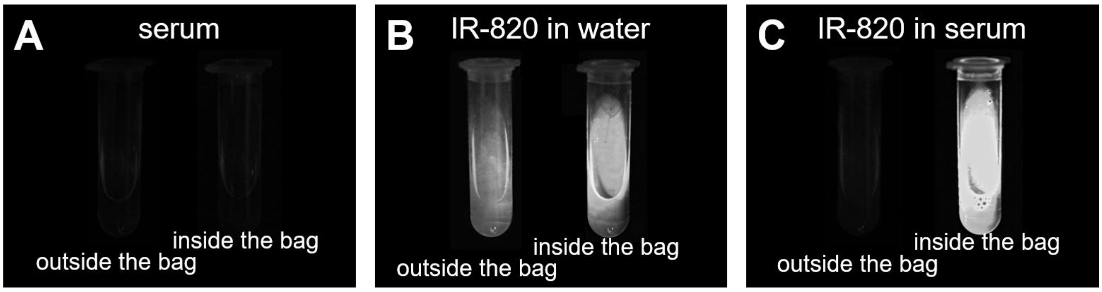

Figure S2. NIR-II fluorescence images of dialyzed **(A)** serum, **(B)** IR-820 in water and **(C)** IR-820 in serum inside the dialysis bags (8 kDa) and outside the dialysis bags (solution passed through the dialysis membrane), under the 793 nm laser (20 mW/cm^2) irradiation. Exposure time: 50 ms. Dialysisi lasted for 24 h.

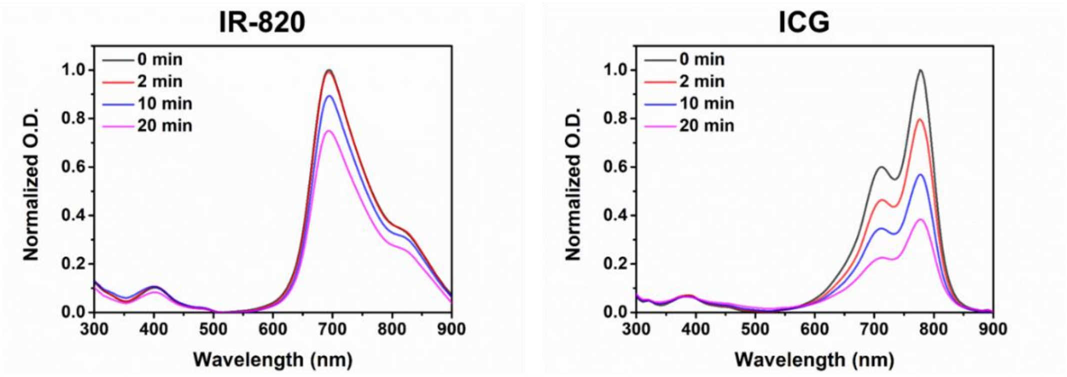

Figure S3. Changes of absorption spectra of IR-820 in serum and ICG in serum, under the 793 nm laser (1.5 W/cm^2) irradiation for 20 minutes.

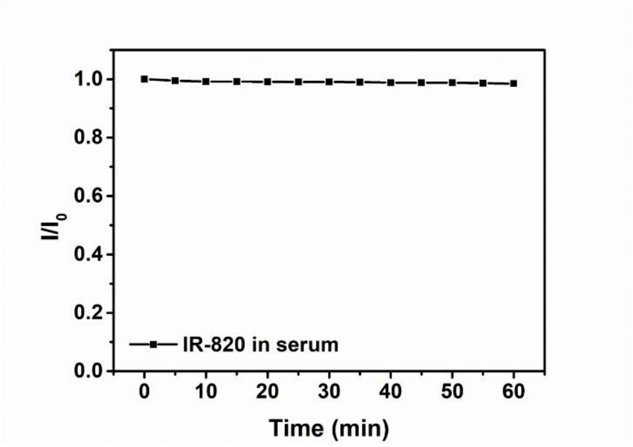

Figure S4. Changes of NIR-II fluorescence intensities of IR-820 in serum under the 793 nm laser (20 mW/cm^2) irradiation for 60 minutes. Exposure time: 50 ms.

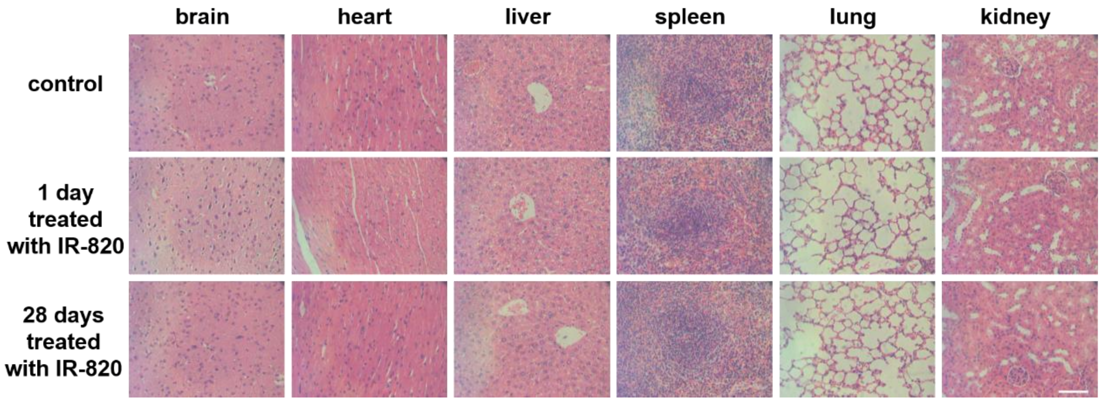

Figure S5. Microscopic images of tissue sections from mice injected with 1 × PBS solution (200 μL) as control, PBS solution of IR-820 (0.5 mg/mL, 200 μL) for 1 day and 28 days respectively. Scale bar: 50 μm.

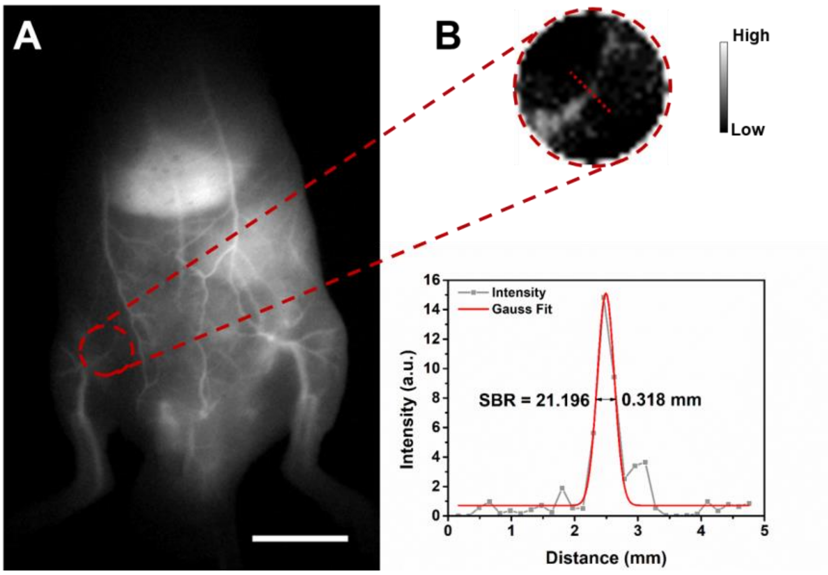

Figure S6. *In vivo* NIR-II fluorescence (beyond 1200 nm) whole-body imaging of a mouse. **(A)** A typical image of the mouse 5 min post the intravenous injection of IR-820 (0.5 mg/mL, 200 μL), taken under 793 nm excitation (15 mW/cm^2). Exposure time: 50 ms. Scale bar: 10 mm. **(B)** A cross-sectional fluorescence intensity profile along the red-dashed line of the mice treated with IR-820. The Gaussian fit to the profile is shown in red line.

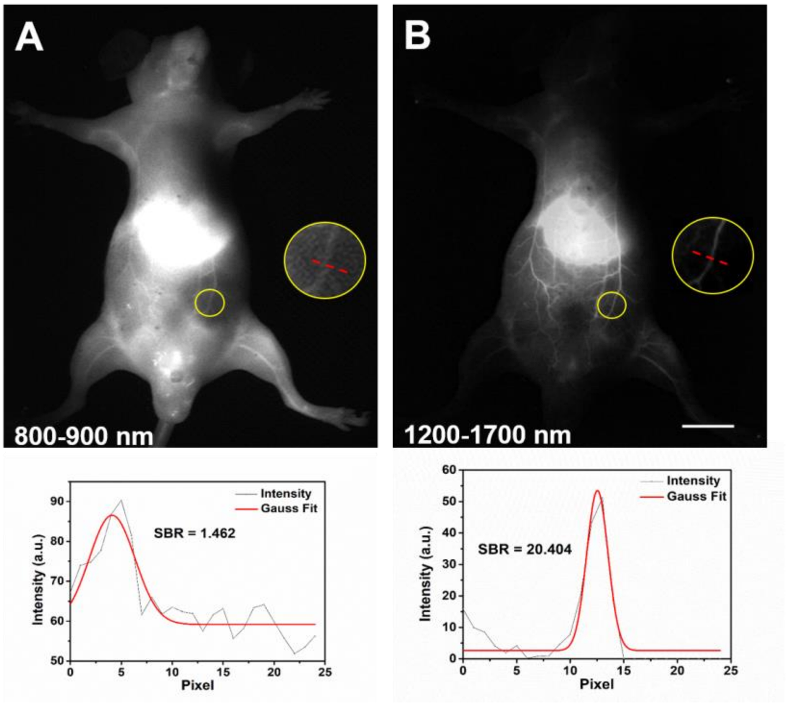

Figure S7. *In vivo* **(A)** NIR-I and **(B)** NIR-II fluorescence images of a mouse post intravenous injection of IR-820 (0.5 mg/mL, 200 μL), under the 793 nm laser (20 mW/cm^2) irradiation. Scale bar: 10 mm.

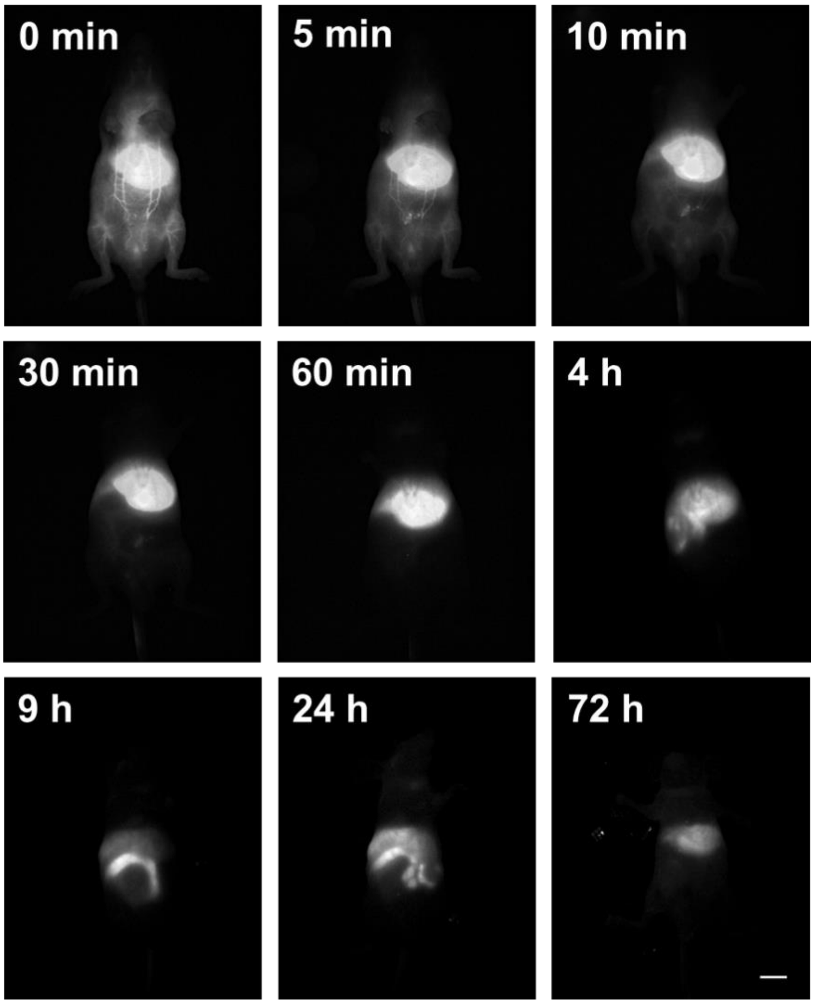

Figure S8. *In vivo* NIR-II fluorescence images of mice at various time points post intravenous injection of IR-820 (0.5 mg/mL, 200 μL), under the 793 nm laser (20 mW/cm^2) irradiation. Exposure time: 50 ms. Scale bar: 10 mm.

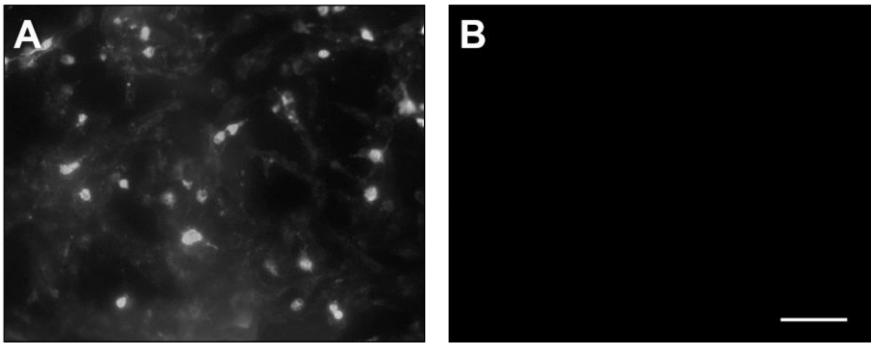

Figure S9. NIR-II fluorescence images of UMUC3 cells treated with **(A)** IR-820 and **(B)** PBS under 793 nm laser excitation (2 W/cm^2). Scale bar: 100 μm.

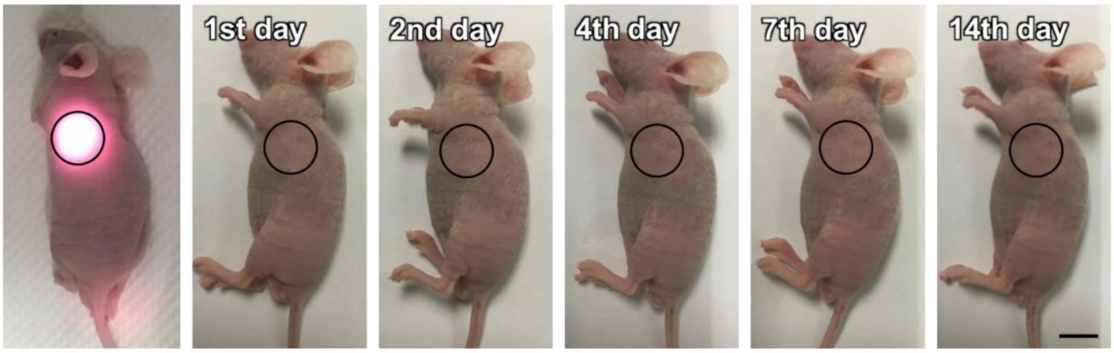

Figure S10. Images of a mouse whose skin was irradiated by the laser (793 nm, 2 W/cm^2) for 10 min, and observed during the next 2 weeks. Scale bar: 10 mm.

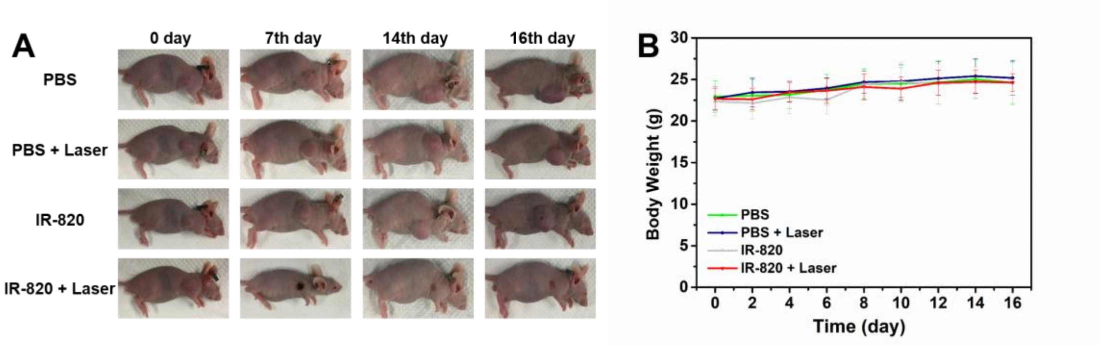

Figure S11. (A) The photographs of tumor-bearing mice, at different time points (0, 7, 14 and 16 days) post various treatments. **(B)** Body weight curves of the tumor-bearing mice after receiving various treatments as a function of time.

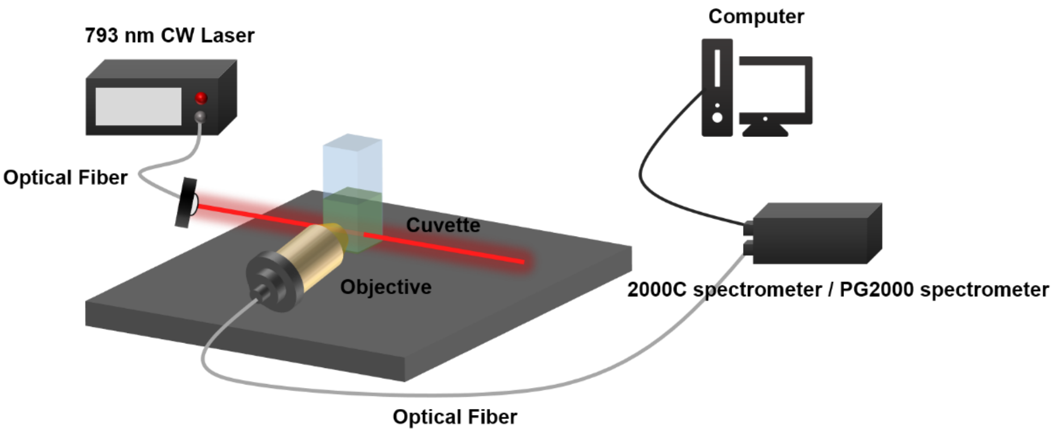

Figure S12. Schematic illustration of the NIR-II fluorescence measurement system.

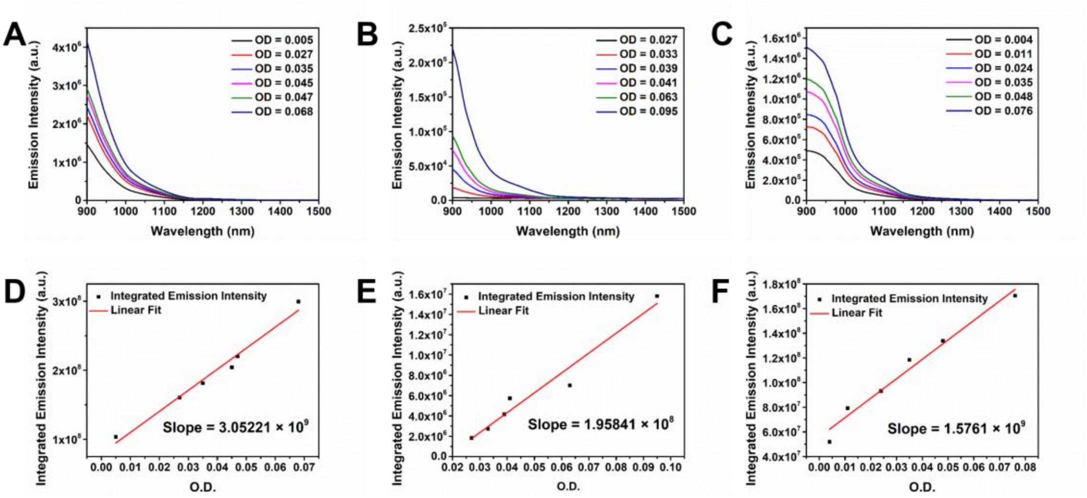

Figure S13. NIR-II fluorescence spectra of **(A)** ICG in DMSO, **(B)** IR-820 in water and **(C)** IR-820 in serum with various optical densities (ODs at 793 nm), under the excitation of a 793 nm laser. Integrated NIR-II fluorescence intensities (900-1500 nm) plotted as a function of OD at 793 nm for **(D)** ICG in DMSO (reference solution), **(E)** IR-820 in water and **(F)** IR-820 in serum. The data were fitted into linear functions with slopes of **(D)** 3.052×10^9 for ICG in DMSO, **(E)** 1.958×10^8 for IR-820 in water and **(F)** 1.576×10^9 for IR-820 in serum. The measured quantum yields of IR-820 in water and IR-820 in serum were 0.313% and 2.521%, respectively.

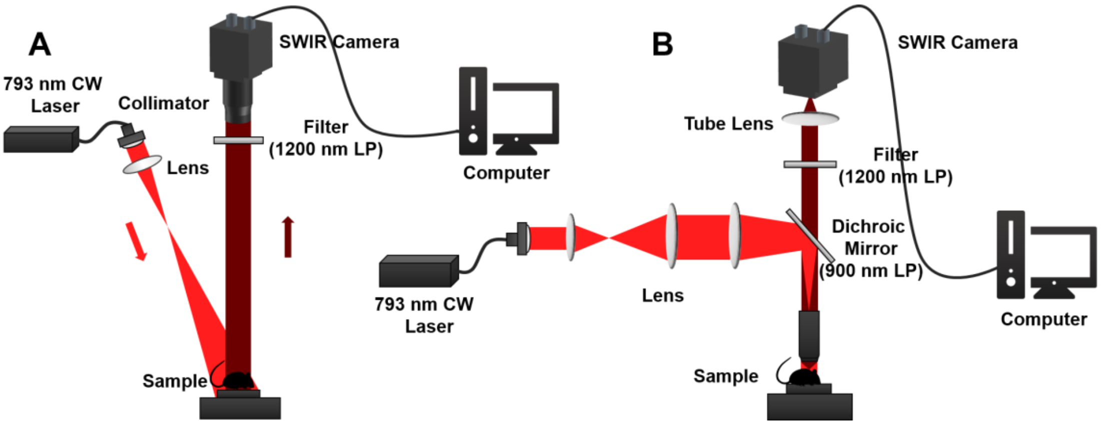

Figure S14. Schematic illustration of the setup for NIR-II fluorescence **(A)** whole-body imaging system and **(B)** microscopic imaging system.

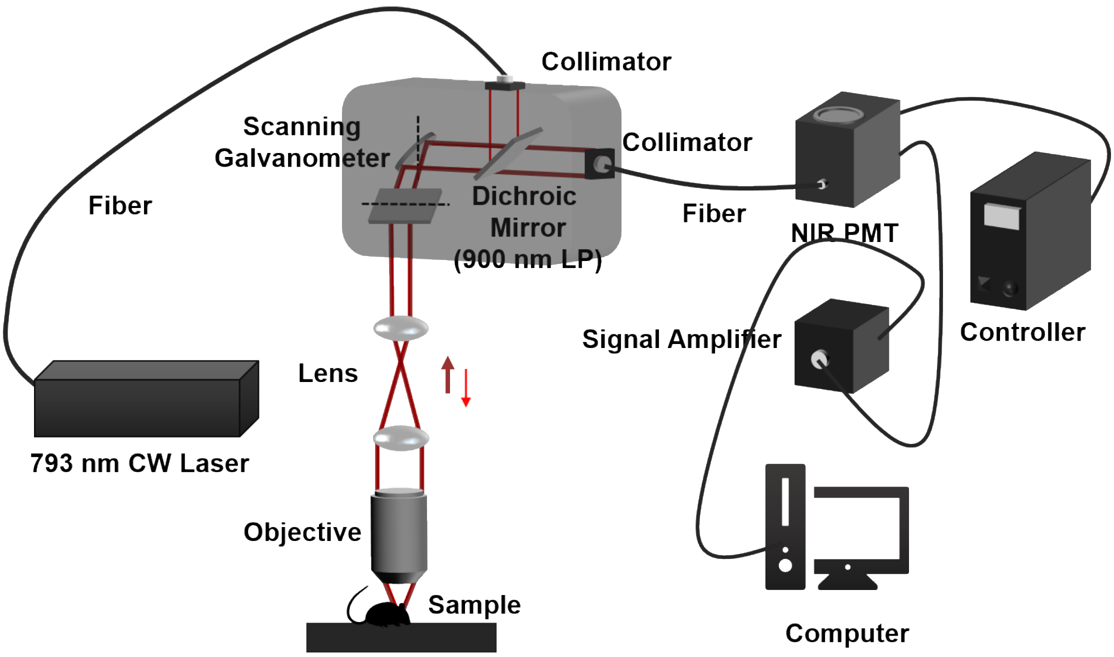

Figure S15. Schematic illustration of the setup for NIR-II fluorescence confocal scanning microscopic imaging system.

Sample	Zeta potential (mV)
serum	-8.05±0.21
IR-820 in water	-0.694±0.015
IR-820 in serum	-10.7±0.3

Table S1. Zeta potentials of serum solution, IR-820 in water and IR-820 in serum.

Test Item	1 d post treatment (n = 3)		28 d post treatment (n = 3)		Reference Range
	Control Group	Experiment Group	Control Group	Experiment Group	
WBC (10^9/L)	3.77±1.29	5.10±1.15	3.23±1.03	2.90±0.85	0.8-6.8
Lymph (10^9/L)	2.67±0.91	4.07±0.68	2.57±1.02	2.37±0.71	0.7-5.7
Mon (10^9/L)	0.13±0.06	0.10±0.10	0.07±0.06	0.00±0.00	0.0-0.3
Gran (10^9/L)	0.97±0.35	0.93±0.42	0.60±0.10	0.53±0.25	0.1-1.8
Lymph% (%)	71.37±3.25	80.47±4.93	77.80±9.90	79.80±5.94	55.8-90.6
Mon% (%)	2.63±0.45	2.17±0.64	2.20±0.70	2.00±0.56	1.8-6.0
Gran% (%)	26.00±3.48	17.37±4.30	20.00±9.20	18.20±5.38	8.6-38.9
RBC (10^{12}/L)	6.96±1.09	6.89±0.95	9.18±0.50	8.85±0.94	6.36-9.42
HGB (g/L)	123.67±20.53	122.67±14.22	152.00±7.81	148.33±16.77	110-143
HCT (%)	40.10±6.46	39.67±4.62	47.60±1.92	48.40±4.85	34.6-44.6
MCV (fl)	57.70±0.26	57.73±1.10	53.87±2.23	54.73±1.32	48.2-58.3
MCH (pg)	17.70±0.26	17.80±0.53	17.17±0.93	16.70±0.44	15.8-19
MCHC (g/L)	307.67±4.04	308.67±6.03	318.67±4.51	305.67±4.16	302-353
RDW (%)	15.57±1.03	14.60±1.04	14.57±1.46	14.27±1.08	13-17
PLT (10^9/L)	870.33±558.46	1251.00±788.34	1025.67±525.90	1652.00±413.29	450-1590
MPV (fl)	5.40±0.10	5.27±0.12	6.17±0.57	6.23±0.67	3.8-6.0

Table S2. Results of blood routine examination for mice. Experimental group represents the mice treated with IR-820 (0.5 mg/mL, 200 μL) and control group represents the mice treated with PBS (1 ×, 200 μL). (n = 3)

Test Item	1 d post treatment (n = 3)		28 d post treatment (n = 3)	
	Control Group	Experiment Group	Control Group	Experiment Group
ALT (U/L)	31.67±4.51	35.00±4.58	30.33±4.51	35.67±7.02
AlkP (U/L)	138.33±5.03	149.00±33.05	89.33±18.23	115.00±7.55
AST (U/L)	86.33±14.64	79.33±6.81	64.33±5.03	93.00±7.55
UA (μmol/L)	77.79±5.51	78.74±27.45	62.35±4.64	76.00±31.24
CRE (μmol/L)	3.44±0.70	6.42±0.93	6.85±0.37	7.56±0.49
UREA (mmol/L)	5.78±0.72	5.23±1.23	8.01±0.69	7.52±0.84

Table S3. Results of hepatic and renal functions test for mice. Experimental group represents the mice treated with IR-820 (0.5 mg/mL, 200 μL) and control group represents the mice treated with PBS (1 ×, 200 μL). (n = 3)

Video S1. Dynamic NIR-II fluorescence cerebrovascular imaging at the depth of 150 μm (objective: 70×). Exposure time: 100 ms.

Video S2. Tracing of the blood flowing in the mouse brain (objective: 25×). Exposure time: 50 ms.
